# Enhanced Bioprinting of 3D Corneal Stroma Patches with Reliability, Assessing Product Consistency and Quality through Optimized Electron Beam Sterilization

**DOI:** 10.1002/adhm.202403118

**Published:** 2025-02-10

**Authors:** Jungbin Yoon, Yeon‐ju Lee, Minji Kim, Ju Young Park, Jinah Jang

**Affiliations:** ^1^ Department of Mechanical Engineering Pohang University of Science and Technology (POSTECH) Pohang 37673 South Korea; ^2^ BioBricks Co., Ltd Pohang 37673 South Korea; ^3^ Department of Convergence IT Engineering POSTECH Pohang 37673 South Korea; ^4^ School of Interdisciplinary Bioscience and Bioengineering POSTECH Pohang 37673 South Korea; ^5^ Institute of Convergence Science Yonsei University Seoul 220‐710 South Korea

**Keywords:** 3D bioprinting, 3D corneal stroma patches, corneal tissue engineering, electron beam sterilization, tissue‐specific bioink

## Abstract

This study focuses on the optimization of sterilization methods for bioprinted three‐dimensional (3D) corneal stroma patches prepared using cornea‐derived decellularized extracellular matrix (Co‐dECM) hydrogels and human keratocytes, with the aim of enhancing clinical applications in corneal tissue engineering. An essential aspect of this study is to refine the sterilization processes, particularly focusing on electron beam (EB) sterilization, to maintain the structural and functional integrity of the Co‐dECM hydrogels while ensuring sterility. The study reveals that EB sterilization outperformed traditional methods like ethylene oxide (EtO) gas and autoclaving, which tend to degrade the biochemical properties of hydrogels. By optimizing the EB‐sterilization process, the essential mechanical and biochemical characteristics needed for successful 3D bioprinting are retained, reducing batch variability in bioprinted 3D corneal stroma patches. Consistency in production is vital for meeting regulatory standards and ensuring patient safety. Moreover, the study investigates the immunomodulatory properties of sterilized hydrogels, emphasizing their potential to minimize inflammatory responses, which is crucial for maintaining keratocyte phenotype. These findings significantly advance biomedical engineering by providing a sterilization method that preserves material integrity, minimizes immunogenicity, and supports the clinical translation of bioprinted corneal stroma patches, offering a promising alternative to donor transplants and synthetic substitutes.

## Graphical abstract

This study optimizes sterilization methods for bioprinted 3D corneal stroma patches made from cornea‐derived decellularized extracellular matrix (Co‐dECM) hydrogels and human keratocytes. It demonstrates the superiority of electron beam sterilization in preserving the mechanical, biochemical, and immunomodulatory properties of hydrogels, ensuring sterility and consistency, which is essential for advancing clinical applications in corneal tissue engineering.

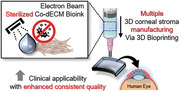

## 1 Introduction

Corneal diseases are a major global health challenge, primarily because of the scarcity of donor cornea and inadequacy of existing synthetic alternatives.^[^ ^1^, ^2^ ^]^ Synthetic corneas could potentially address the significant global challenge of visual impairment caused by corneal diseases, which affect millions and contribute to corneal opacity as the fourth leading cause of blindness worldwide, by substituting scarce donor corneas—only available to about 1 in 70 patients in need—reducing the risk of immune rejection and providing consistent transplant quality for all patients.^[^ ^3^ ^]^ However, the implementation of synthetic corneas is not without significant obstacles. Key issues include low biocompatibility, which can lead to poor integration with the natural tissue of hosts, high cost of production, and limited accessibility, particularly in resource‐limited settings.^[^ ^4^ ^]^ These challenges necessitate further research and development to make synthetic corneas a viable and widely accessible solution.

Three‐dimensional (3D) bioprinting technology presents a transformative approach to creating corneal substitutes by utilizing tissue‐derived decellularized extracellular matrix (dECM) components for enhanced tissue‐specific compatibility and functionality.^[^ ^5^, ^6^ ^]^ This innovative technology involves the use of cornea‐derived dECM (Co‐dECM) hydrogels combined with human keratocytes to fabricate bioprinted 3D corneal stroma patches.^[^ ^7^ ^]^ The Co‐dECM bioink, a specially formulated bioink designed for bioprinting applications, is known for its superior biocompatibility and maintains post‐bioprinting clarity, which is crucial for corneal applications.^[^ ^8^ ^]^ Furthermore, 3D bioprinting technology allows precise control over the production process, enabling customization of corneal stromal patches to meet specific patient needs. This scalability and customization contribute to the increased availability and enhanced suitability of corneal substitutes, making them practical alternatives to traditional transplants.^[^ ^9^ ^]^ These stromal patches are designed not only to repair damaged corneal tissue but also to potentially reduce reliance on donor corneas, which have limited availability, thereby addressing significant challenges in corneal transplantation and tissue repair.

Recent advances in 3D bioprinting for corneal tissue engineering have expanded its potential. For example, the development of bioartificial corneas using electrospun micro‐/nanofibrous decellularized extracellular matrix (dECM) and digital light processing (DLP) 3D bioprinting has demonstrated significant improvements in mechanical properties and cell compatibility. These bioartificial corneas showed enhanced corneal regeneration in animal studies, supporting their potential as alternatives to lamellar keratoplasty.^[^ ^5^ ^]^ Similarly, electrospun gelatin nanofiber membranes have been successfully used as carriers for corneal endothelial cell (CEC) transplantation, which addresses the global shortage of donor corneas.^[^ ^10^ ^]^ Moreover, visible light‐crosslinkable bioinks utilizing advanced photoinitiators and multilength networks have enabled the fabrication of optically transparent, cytocompatible, and structurally precise artificial corneal scaffolds.^[^ ^11^ ^]^ These innovations demonstrate the growing versatility and efficacy of 3D bioprinting technologies in corneal tissue engineering.

Transitioning tissue‐derived dECM‐based bioprinting from research laboratories to clinical settings involves addressing significant challenges, particularly sterilization and safety protocols.^[^ ^12^ ^]^ The regulation of biopharmaceutical manufacturing is stringent, aiming to ensure product safety and efficacy.^[^ ^13^ ^]^ This involves comprehensive quality control, hygiene management, and validation of material safety, especially when xenogeneic materials are used.^[^ ^14^ ^]^ Biopharmaceutical manufacturing must comply with several regulatory conditions, including Good Manufacturing Practices (GMP) to ensure consistent product quality, rigorous testing and safety assessments of raw materials, process validation at each manufacturing stage, and stringent hygiene‐management protocols.^[^ ^15^ ^]^

To ensure that each unit of the bioprinted product is free of viable microorganisms, it is essential to achieve a specific sterility assurance level (SAL).^[^ ^16^, ^17^ ^]^ Sterilization processes must effectively reduce bioburden while preserving the safety and viability of bioprinted 3D patches for clinical applications.^[^ ^18^, ^19^ ^]^

The current lack of clear standards for evaluating the quality of bioprinted biopharmaceutical products poses a significant challenge. Given the specific safety issues associated with xenogeneic materials and the absence of standardized quality‐evaluation criteria, it is imperative to establish comprehensive Chemistry, Manufacturing, and Controls (CMC) standards.^[^ ^20^, ^21^, ^22^ ^]^ International cooperation among regulatory bodies is essential to maintaining regulatory consistency and enhancing the overall efficiency of biopharmaceutical regulation. Additionally, minimizing batch‐to‐batch variation is crucial for the translation of 3D bioprinting technology from the lab to the clinic. Batch consistency is crucial for ensuring safe, effective, and reproducible results, which are essential for gaining regulatory approvals, facilitating standardization, and maximizing the number of products that pass quality control checks.^[^ ^23^, ^24^ ^]^ Regulatory bodies, such as the Food and Drug Administration (FDA), demand evidence of consistent quality for product approval.^[^ ^25^ ^]^ Uniformity not only enhances patient safety and minimizes risks associated with implantation but also supports precise research outcomes, reduces waste, and helps make advanced treatments more accessible and affordable.^[^ ^26^ ^]^

There are three prominent sterilization techniques, each with its distinct advantages and limitations. Electron Beam (EB) irradiation employs high‐energy electrons for rapid sterilization without damaging sensitive biomaterials, making it ideal for delicate 3D bioprinted products.^[^ ^27^ ^]^

Autoclaving uses high‐pressure saturated steam to sterilize heat‐resistant materials effectively without the use of toxic chemicals, making it environmentally friendly and cost‐effective.^[^ ^28^ ^]^ The choice between these methods depends on balancing efficacy, safety, material compatibility, and regulatory compliance. Ethylene oxide (EtO) exposure is highly effective for heat‐ and moisture‐sensitive materials and offers deep penetration into complex devices.^[^ ^29^ ^]^ Despite its widespread use, EtO gas is associated with substantial safety and environmental concerns.^[^ ^30^ ^]^

Recently, the European Union has proposed a ban on the use of EtO gas for sterilization because of its carcinogenicity and potentially harmful residues that can remain on sterilized materials.^[^ ^31^, ^32^ ^]^ These regulatory changes highlight the urgent need for alternative sterilization methods to ensure the safety and effectiveness of biopharmaceutical products without the risks associated with EtO gas.^[^ ^33^ ^]^

This study discusses the critical importance of selecting appropriate sterilization techniques to maximize the therapeutic benefits of bioprinted 3D corneal stromal tissue. Specifically, we focused on optimizing the sterilization process for Co‐dECM, which is used in creating multiple 3D corneal stroma patches. This optimization aimed to enhance the safety of these patches and ensure batch‐to‐batch consistency, which is a key factor for reliable quality and successful clinical application. This study represents a significant breakthrough in biomedical engineering, contributing to the field of ophthalmic tissue engineering by optimizing sterilization techniques, thereby enhancing the quality and safety of bioprinted 3D corneal stroma patches and advancing their practical application as a new treatment option for individuals with corneal blindness.

## 2 Results and Discussion

### 2.1 Impact of Various Sterilization Methods on the Biochemical and Rheological Properties of dECM Materials for 3D Bioprinting Applications

#### 2.1.1 Impact of Various Sterilization Techniques on Biochemical Properties of Cornea dECM Materials

**Figure 1:**
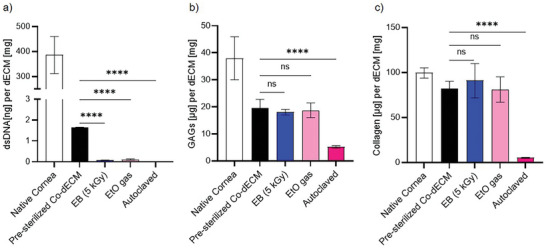
Biochemical composition analysis of different corneal matrices. a) Double‐stranded DNA (dsDNA) content per decellularized extracellular matrix (dECM) in mg. b) Glycosaminoglycans (GAGs) content measured in µg per dECM. c) Collagen content quantified in µg per dECM. Statistical significance is indicated by asterisks (****p < 0.0001) and “ns” (*not significant*). Error bars represent standard deviation. Samples analyzed include native cornea, pre‐sterilized Co‐dECM, EB (5 kGy), EtO gas, and autoclaved preparations.

Initially, we assessed the biochemical integrity of Co‐dECM materials (Figure S1, Supporting Information) following exposure to different sterilization methods: a 5 kGy dose of EB irradiation, EtO gas exposure, and autoclaving. The visual appearance of Co‐dECM materials sterilized using EB and EtO gas showed no significant differences compared to the pre‐sterilized group (which was only sterilized with a 1% peracetic acid solution during Co‐dECM preparation). In contrast, autoclaving caused noticeable shrinkage and yellow discoloration of the Co‐dECM materials (Figure S2, Supporting Information). Biochemical assays revealed that both EB and EtO gas sterilization effectively maintained controlled contamination levels, successfully reducing the residual double‐stranded DNA (dsDNA) content while preserving the glycosaminoglycan (GAGs) and collagen contents (**Figure** **1**a,b,c). This preservation is crucial for maintaining the biological and mechanical properties of dECM‐derived bioinks, such as hydrogels and cellular components, which enhance cellular activity and support tissue development. In contrast, autoclaving resulted in severe reductions in GAGs and collagen levels, compromising the functional integrity of the material.

EB irradiation and EtO gas exposure effectively reduced residual double‐stranded DNA (dsDNA) content through distinct mechanisms (Figure 1a). EB irradiation employs high‐energy electrons to induce structural damage, including single‐ and double‐strand breaks, in dsDNA molecules. This disruption renders dsDNA biologically inactive, reducing the risk of immune rejection in implanted materials.^[^ ^34^, ^35^ ^]^ EB irradiation achieves uniform dsDNA reduction without high temperatures or chemical agents, thereby preserving the structural integrity of the dECM.^[^ ^36^ ^]^ In comparison, EtO gas sterilization occurs via chemical alkylation, which modifies nucleic and amino acids. This prevents normal base pairing and replication, neutralizing the residual biological activity of dsDNAs.^[^ ^37^ ^]^ The penetration of EtO gas into the intricate material structures ensures comprehensive sterilization, making it a reliable method for reducing dsDNA levels while maintaining material functionality.

The autoclaved Co‐dECM materials exhibited the lowest levels of residual dsDNA (Figure 1a). This reduction is attributable to the destruction of nucleic acids, including dsDNA, under high heat and pressure used during autoclaving. However, this method significantly compromised the structural and functional integrity of the Co‐dECM components, as evidenced by the dramatically reduced levels of GAGs and collagen (Figure 1b,c). Temperatures ≈121 °C, generally used for autoclaving, can denature collagen, the primary structural protein in the dECM, disrupting its triple‐helical structure and diminishing its biomechanical properties.^[^ ^19^, ^38^ ^]^ This denaturation can adversely affect the ability of the printed scaffolds to support cell attachment and proliferation. Consequently, autoclaving is deemed unsuitable for sterilizing Co‐dECM materials intended for use in fabricating 3D corneal stroma patches, because of the detrimental effects of high temperatures and moisture on these materials. Given these adverse effects, alternative sterilization methods such as EB irradiation and EtO gas exposure are considered more appropriate. These alternatives strike a balance between ensuring sterility and preserving the biochemical and structural properties of dECM materials.

To achieve a balance between sterilization efficacy and material integrity, a 5 kGy EB dose was selected based on extensive preliminary studies evaluating various irradiation doses. Lower doses, such as 1 kGy, were insufficient for reliable sterilization, as indicated by inadequate visual confirmation using exposure indicators (Figure S3, Supporting Information). The higher doses, such as 30 kGy—commonly used for sterilizing durable materials—resulted in significant degradation of critical biochemical properties, including collagen and GAG.^[^ ^39^, ^40^ ^]^ These higher doses also inhibited the formation of functional dECM‐derived hydrogels, further limiting their suitability for biofabrication.^[^ ^18^ ^]^ Gamma radiation, another commonly used sterilization method, typically requires a minimum dose of 25 kGy. However, the interaction mechanisms of gamma photons and EB electrons with biological materials differ fundamentally, complicating direct dose equivalence between the two methods.^[^ ^41^ ^]^

In our study, a 5 kGy EB dose effectively sterilized Co‐dECM materials while preserving their essential biochemical and structural properties for biofabrication (Figure 1 and Figure S4, Supporting Information). EB sterilization, which is a high‐energy ionizing radiation method, induces protein chain scission and generates free radicals that can oxidize amino acid side chains (e.g., methionine and cysteine) and cause the deamidation of asparagine and glutamine residues. These modifications can alter protein charge, folding stability, and biological activity.^[^ ^42^, ^43^ ^]^ From the circular dichroism (CD), the spectra of pre‐sterilized Co‐dECM and EB (5 kGy) show a negative peak ≈208–210 nm and a small shoulder at 222 nm, which is indicative of α‐helical contents (Figure S4, Supporting Information). A similar shoulder near 222 nm reflects contributions from triple‐helical structures, which are characteristic of collagen.^[^ ^44^, ^45^ ^]^ These findings suggested a well‐preserved structure with organized secondary features, which likely represent the native state of the ECM. In the case of EB irradiated at 30 kGy, the negative peak near 200 nm becomes significantly deeper, characteristic of an increase in the random coil content (Figure S4, Supporting Information). This indicated substantial structural disruption, denaturation, or unfolding caused by high‐dose electron beam exposure. In conclusion, α‐helices and triple‐helical structures are sensitive to electron beam exposure, with higher doses causing significant disruption. The observed increase in random coil content with higher doses reflects the unfolding and loss of structural integrity. For applications requiring structural preservation (e.g., tissue engineering or biomedical scaffolds), lower doses (e.g., 5 kGy) are preferable because higher doses (30 kGy) result in severe degradation.

Among the sterilization methods tested, EB irradiation at an optimized 5 kGy dose emerged as the most suitable technique for Co‐dECM materials. It provided effective sterilization while preserving essential biochemical properties, making it ideal for biofabrication. EtO gas also proved effective, maintaining material integrity and offering a viable alternative. However, autoclaving was deemed unsuitable due to its detrimental effects on the biochemical and structural properties of Co‐dECM materials.

### 2.2 Evaluation of Sterilization Techniques for Co‐dECM Hydrogels and Their Impact on Rheological Properties for 3D Bioprinting Applications

**Figure 2:**
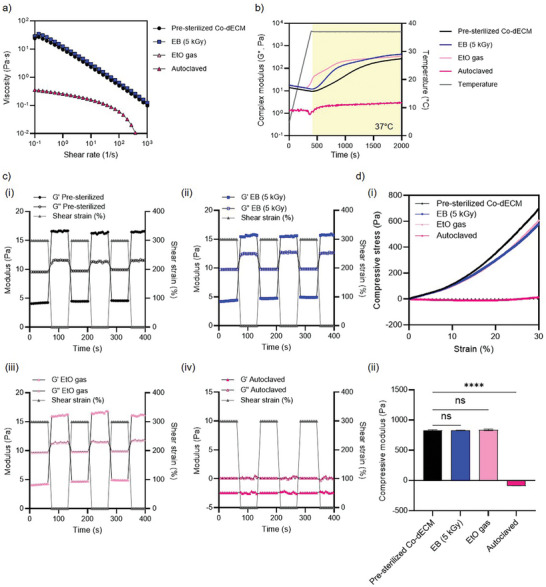
Rheological and mechanical characterization of sterilized corneal matrices. a) Viscosity measurements as a function of shear rate for differently sterilized Co‐dECM samples. b) Complex modulus changes over time during temperature‐controlled gelation at 37 °C. c) Step strain measurements showing storage (G') and loss (G″) moduli with applied shear strain for: i) Pre‐sterilized Co‐dECM, ii) EB (5 kGy) treated samples, iii) EtO gas‐treated samples, and iv) autoclaved samples. d) Mechanical properties under compression: i) Compressive stress‐strain curves up to 30% strain ii) Quantitative comparison of compressive modulus across different sterilization methods. Statistical significance is indicated by asterisks (****p < 0.0001) and “ns” (*not significant*). Error bars represent standard deviation.

Based on the methodology described in a previous study, we prepared 2.0% Co‐dECM hydrogels incorporating 0.5 M acetic acid, distilled water, and pepsin.^[^ ^8^ ^]^ We further investigated the effects of three sterilization techniques‐ EB irradiation (5 kGy), EtO gas exposure, and autoclaving‐on the rheological and mechanical properties of 2.0% Co‐dECM hydrogels designed for 3D bioprinting applications. These hydrogels, intended for use as bioinks incorporating human keratocytes, were evaluated for their viscosity, thermal gelation behavior, shear recovery properties, and compressive modulus to assess their suitability for 3D bioprinting technology (**Figure** **2**).

In terms of viscosity, the results demonstrated that Co‐dECM hydrogels sterilized by EB (5 kGy) irradiation and EtO gas exposure retained their viscosity, maintaining their suitability for 3D bioprinting technology (Figure 2a). In contrast, autoclaving caused a significant reduction in viscosity, rendering the hydrogel unsuitable for extrusion‐based printing (Figure 2a). Reduced viscosity negatively impacts printability, as it compromises the ability of dECM‐derived hydrogels to flow smoothly through the cylindrical printing nozzle and prevents proper layer formation. Maintaining optimal viscosity is critical for extrusion‐based 3D bioprinting techniques to achieve consistent deposition and ensure structural integrity.^[^ ^46^, ^47^ ^]^

The thermal gelation behaviors of the hydrogels were analyzed. Pre‐sterilized, EB (5 kGy)‐sterilized, and EtO‐sterilized Co‐dECM hydrogels exhibited an increase in modulus when the temperature was raised from 4 to 37 °C, indicating effective thermal gelation (Figure 2b). However, autoclaving disrupted this gelation process, as evidenced by the absence of an increase in modulus (Figure 2b). This implies that autoclaving damages the structural integrity of the hydrogels, impairing their ability to gel under physiological conditions, which is crucial for 3D bioprinting applications.

Shear recovery behavior, which evaluates the ability of the hydrogel to recover its structure after being subjected to stress, was assessed for all samples (Figure 2c).^[^ ^48^ ^]^ Pre‐sterilized, EB (5 kGy)‐sterilized, and EtO‐sterilized Co‐dECM hydrogels demonstrated effective shear recovery, as indicated by their storage modulus (G') and loss modulus (G’) behaviors (Figure 2c (i–iii)). This property is essential for restoring the structural integrity of Co‐dECM hydrogels after extrusion stress and maintaining the fidelity of the printed 3D structures. However, the autoclaved Co‐dECM hydrogels exhibited a significantly impaired shear recovery behavior, further emphasizing their unsuitability for 3D bioprinting applications (Figure 2c (iv)).

Finally, the compressive stress–strain curves revealed that the pre‐sterilized, EB (5 kGy)‐sterilized, and EtO‐sterilized Co‐dECM hydrogels maintained their mechanical integrity, with consistent compressive modulus (Young's modulus) values within the 0–5% strain range (Figure 2d). Conversely, autoclaving caused a drastic reduction in the compressive modulus, thereby indicating a severe degradation of the mechanical properties (Figure 2d). This loss of stiffness compromises the ability of the Co‐dECM hydrogel to support load‐bearing 3D structures, which is a critical requirement for tissue engineering and 3D bioprinting applications.^[^ ^38^ ^]^

For suturability, our bioprinted 3D corneal stromal patches demonstrated sufficient structural integrity to withstand suturing. The optimized EB sterilization process preserved collagen content and crosslinking within the Co‐dECM hydrogels, thereby providing tensile strength necessary for suture retention. However, further optimization may be required to match the suture‐holding capacity of the native corneal tissue. Future studies must include specific suture retention tests for quantifying and improving this property.^[^ ^3^ ^]^ Rheological and compression tests (Figure 2) confirmed that the EB‐sterilized constructs maintained a compressive modulus comparable to that of pre‐sterilized samples within the physiologically relevant 0–5% strain range.^[^ ^49^ ^]^ This suggests that our bioprinted constructs possessed mechanical properties suitable for handling and potential implantation.^[^ ^50^ ^]^ However, the stiffness of these constructs may not fully replicate that of the native corneal stroma. Future efforts are expected to focus on refining the bioink composition and cross‐linking strategies to better mimic native tissue mechanics.^[^ ^51^, ^52^ ^]^

In conclusion, these findings emphasize the detrimental effects of autoclaving on the rheological and mechanical properties of 2.0% Co‐dECM hydrogels, thereby rendering them unsuitable for sterilization. In contrast, EB (5 kGy) irradiation and EtO sterilization preserved key properties such as viscosity, viscoelasticity, and compressive strength, ensuring their suitability for 3D bioprinting applications.^[^ ^53^, ^54^ ^]^ These results highlight the importance of selecting sterilization methods that maintain the structural and functional integrity of Co‐dECM hydrogels, which is crucial for reliable bioprinting outcomes and cell viability.^[^ ^55^ ^]^

Our study supports the use of EB (5 kGy) irradiation and EtO gas as preferable sterilization methods that meet the stringent rheological demands required for manufacturing bioink for the formation of advanced biomedical applications, such as bioprinted 3D corneal stroma.^[^ ^56^, ^57^, ^58^ ^]^ In particular, EtO gas is an effective sterilization agent that is widely used in medical devices that cannot withstand high temperatures.^[^ ^44^ ^]^ However, its use has raised significant safety concerns because of its toxicity.^[^ ^45^ ^]^ EtO is classified as a human carcinogen by the International Agency for Research on Cancer (IARC) and the U.S. Environmental Protection Agency (EPA). EtO gas exposure can occur during sterilization or from residual gas in sterilized items. Proper aeration is crucial for dissipating the absorbed gas; however, improper aeration can leave residual EtO, leading to major health risks such as leukemia, and stomach and pancreatic cancer from chronic exposure, and respiratory and neurological effects from acute exposure.^[^ ^46^ ^]^ Additionally, EtOH can react with body tissues, causing inflammatory and allergic responses.^[^ ^59^, ^60^ ^]^ EtO can also form harmful byproducts, such as ethylene glycol, upon reacting with environmental elements.^[^ ^61^ ^]^ Owing to these risks, regulatory bodies enforce strict guidelines on EtO use, including exposure controls and thorough aeration.^[^ ^62^ ^]^

Ultraviolet (UV) sterilization is presented as a method that induces pyrimidine dimer formation in nucleic acids, limiting microbial activity. However, the penetration depth was minimal, as reported previously.^[^ ^63^ ^]^ Protein damage under UV irradiation is limited to surface‐level effects, with a minimal impact on bulk material properties.^[^ ^64^ ^]^ Rheological measurements revealed that UV treatment had a minimal effect on the gelation kinetics and mechanical properties of Co‐dECM hydrogels, which further underscore its suitability as a sterilization method without compromising functional integrity (Figure S5a,b,c). UV irradiation did not induce significant alterations in the shear recovery behavior or compressive stress properties of the Co‐dECM hydrogels, which shows similar mechanical behavior within the linear range (0–5% strain), thereby indicating that UV sterilization did not adversely affect the mechanical integrity of the hydrogel (Figure S5d,e). Further, the circular dichroism (CD) spectra demonstrated minimal disruption of the secondary structures of the Co‐dECM proteins following UV treatment (Figure S5f, Supporting Information). These findings highlight the milder effects of UV sterilization on the structural and functional integrity of Co‐dECM materials, which is consistent with the primary action of UV on nucleic acids and limited penetration into the material bulk.^[^ ^64^ ^]^ However, UV irradiation may reach only the surface of the material without fully penetrating it. Consequently, the sterilization process may be incomplete, which limits its ability to ensure the safety of the materials for clinical applications. Although UV sterilization is a less destructive method, its limitations highlight the need for additional or alternative strategies, such as EB irradiation, which is often preferred for sterilizing Co‐dECM materials to enhance safety in biomedical applications.^[^ ^65^ ^]^

We previously mentioned that dECM materials were exposed to a relatively lower EB dose than the usual dose of 25–30 kGy used for medical devices.^[^ ^16^ ^]^ Hence, additional sterility tests were conducted to confirm the sterilization of the dECM materials when treated with low EB exposure (5 kGy). A comprehensive sterility assessment protocol adhering to ISO standards was developed and implemented. These tests included bioburden, endotoxin, and sterility testing. The protocol was applied to both controls before sterilization and EB (5 kGy)‐sterilized Co‐dECM materials (**Table** **1**). Bioburden testing of the pre‐sterilized Co‐dECM material indicated minimal bacterial presence, with aerobic bacteria detected in only one out of 10 samples (Table 1, top column). Endotoxin testing of the EB (5 kGy)‐sterilized Co‐dECM materials demonstrated compliance with the stringent safety thresholds prescribed by The United States Pharmacopeia. The permissible endotoxin limit for general medical device usage is set at less than 20 EU unit^−1^.^[^ ^66^ ^]^ For devices intended for use in neurologically sensitive areas, such as the brain and spinal cord, the limit is more restrictive at less than 2.15 EU unit^−1^.^[^ ^67^ ^]^ The endotoxin levels in EB (5 kGy)‐sterilized Co‐dECM samples were 2.198 ± 0.805 EU mL^−1^, thereby satisfying the necessary standards for broader medical applications (Table 1, middle column). Sterility tests compliant with ISO 11737‐2:2019 standards indicated no microbial growth in the EB (5 kGy) sterilized materials, confirming the effectiveness of the EB sterilization process (Table 1, bottom column).^[^ ^68^ ^]^ This comprehensive assessment not only verified the microbiological safety of EB (5 kGy) sterilized Co‐dECM materials but also underscored their suitability for clinical use, especially in manufacturing corneal substitutes. The results also highlight that even though the EB sterilization doses were relatively low, the sterilization was still effective. This demonstrates the capability of EB sterilization to produce dECM materials that meet the high safety and functionality standards essential for tissue engineering and regenerative medicine.

**Table 1:** Microbiological Assessment of Co‐dECM Pre‐ and Post‐EB Sterilization.

Test Method	Application	Result
Bioburden Test	Co‐dECM materials before sterilization (*n* = 10)	Average 0.15 CFU unit^−1^; minimal bacterial presence detected (Only one aerobic bacteria was found in 10 samples).
Endotoxin Test	EB (5 kGy)‐sterilized Co‐dECM material (0.1 g each, n = 3)	Mean endotoxin value: 2.198 ± 0.805 EU mL^−1^.
Sterility Test	EB (5 kGy)‐sterilized Co‐dECM material (*n* = 3)	No microbial growth was detected according to ISO 11737‐2:2019.

### 2.3 Manufacturing 3D Corneal Stroma Patches with Minimal Batch‐to‐Batch Variation, Ensuring Sustainable Quality

#### 2.3.1 Optimization of Printing Parameters for Hydrogels Prepared Using EB‐Sterilized and Pre‐Sterilized Co‐dECM

**Figure 3:**
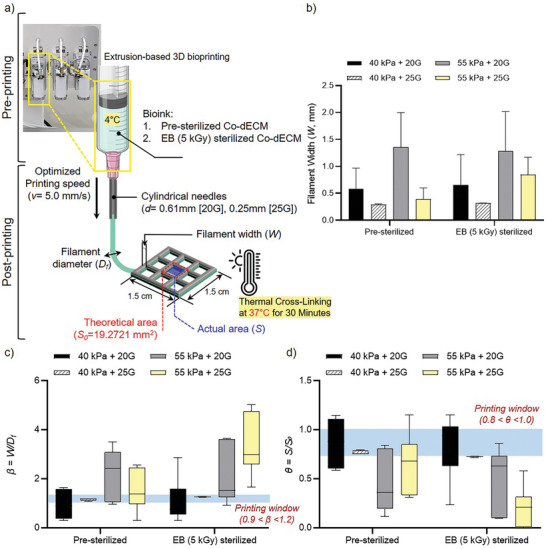
Extrusion‐based 3D bioprinting parameters and filament characterization. a) Schematic of the extrusion‐based 3D bioprinting process showing a pre‐printing setup at 4 °C, bioink compositions, and post‐printing parameters. A comprehensive 3D bioprinting process workflow begins with the pre‐printing preparation at 4 °C, where two distinct bioink formulations are prepared: Pre‐sterilized Co‐dECM and EB (5 kGy) sterilized Co‐dECM. The printing process employs an optimized printing speed of 5.0 mm s^−1^ through cylindrical needles of two different specifications: 20G with a 0.61 mm diameter and 25G with a 0.25 mm diameter. The post‐printing phase involves the production of a 1.5 cm × 1.5 cm grid structure, where both filament width (*W*) and diameter (*D_f_*) are measured. The printed structure maintains a theoretical area (*S_0_*) of 19.2721 mm^2^, with the actual printed area (*S*) measurements taken for comparison. The process concludes with a thermal cross‐linking step conducted at 37 °C for a duration of 30 min to stabilize the printed construct. b) Filament width (*W*) measurements under different printing conditions using combinations of pressure (40 kPa or 55 kPa) and needle gauges (20G or 25G) for Pre‐sterilized and EB (5 kGy) sterilized Co‐dECM. Error bars represent standard deviation. c) *β* ratio (*W/D_f_*) analysis across different printing parameters, with a printing window defined between 0.9 < *β* < 1.2. d) *θ* ratio (*S/S_0_*) analysis for different printing conditions, with the optimal printing window between 0.8 < *θ* < 1.0.

To ensure the reproducibility of 3D corneal stroma patches as viable substitutes for the human cornea, we optimized printing parameters to minimize variability and enhance consistency (**Figure** **3**). Hydrogels were prepared using 2.0% Co‐dECM, sterilized either via electron beam (EB, 5 kGy) or pre‐sterilization, and processed using an extrusion‐based 3D bioprinting system. During printing, the bioink was maintained at 4 °C, and cylindrical needles with diameters of 0.61 mm (20G) and 0.25 mm (25G) were utilized. Key parameters, including filament width (*W*), filament diameter (*D_f_*), aspect ratio (*β = W/D_f_*), and area ratio (*θ = S/S₀*), were systematically evaluated under various printing conditions.^[^ ^69^, ^70^, ^71^, ^72^, ^73^ ^]^ A 1.5 × 1.5 cm grid pattern was printed to assess the structural and functional integrity of the constructs (Figure 3).

We systematically varied the printing speed (2.5, 5.0, and 10.0 mm s^−1^) and extrusion pressure (40 and 55 kPa) to determine the optimal printing conditions. Filament width (*W*) analysis showed that a speed of 5.0 mm s^−1^ produced a uniform filament morphology with minimal variability (Figure S6a,b, Supporting Information). At this speed, a balance was achieved between material deposition and structural integrity, which resulted in well‐defined and reproducible grid structures without distortion. Slower speeds (2.5 mm s^−1^) caused excessive material deposition, thereby leading to filament spreading and sagging, which resulted in high aspect ratio (*β*) values (Figure S6c,d, Supporting Information). Conversely, faster speeds (10.0 mm s^−1^) caused under‐extrusion and filament breakage, producing thinner and inconsistent filaments with low β values. The β values at 5.0 mm s^−1^ consistently fell within the optimal “printing window” (*0.9 < β* < *1.2*), confirming it as the most reliable speed for structural consistency and reproducibility (Figure S6c,d, Supporting Information).

The influence of cylindrical printing nozzle diameter and extrusion pressure was also investigated to optimize printing resolution and structural integrity. Smaller cylindrical printing nozzles (25G, 0.25 mm) provided higher resolution but required higher pressures (55 kPa), increasing the risk of clogging and potentially compromising cell viability. In contrast, larger cylindrical printing nozzles (20G, 0.61 mm) operated at lower pressures but resulted in reduced resolution. The optimal configuration involved the use of a 25G cylindrical printing nozzle with an extrusion pressure of 40 kPa, which produced uniform filament widths and maintained high structural fidelity while minimizing clogging risks (Figure 3b). To ensure consistency and printability, aspect ratio (*β*) and area ratio (*θ*) values were closely monitored. The *β* value, representing the ratio of filament width (*W*) to diameter (*D_f_*), was consistently maintained within the optimal range (0.9–1.2), ensuring structural stability across both pre‐sterilized and EB (5 kGy) sterilized Co‐dECM hydrogels (Figure 3c). Similarly, the area ratio (*θ = S/S₀*), which evaluates bioink spreading behavior, remained within the desired range (0.8–1.0) under optimal conditions (Figure 3d). These results demonstrated effective control over bioink deposition, enabling the reproducibility of 3D printed constructs.

A comparative analysis of pre‐sterilized and EB (5 kGy) sterilized Co‐dECM hydrogels revealed minimal differences in filament morphology and printing performance, confirming that both sterilization methods are suitable for producing consistent and reproducible 3D bioprinted corneal stroma patches. These optimized printing parameters form the basis for a standardized protocol, supporting the development of standard operating procedures (SOPs) to ensure uniformity and reliability across all bioprinted products.^[^ ^51^, ^74^, ^75^, ^76^ ^]^ This advancement provides a robust foundation for enhancing reproducibility and structural integrity in future biomedical applications.^[^ ^77^ ^]^

#### 2.3.2 Manufacturing Multiple Batches of 3D Corneal Stroma Patches with Controlled Parameters

**Figure 4:**
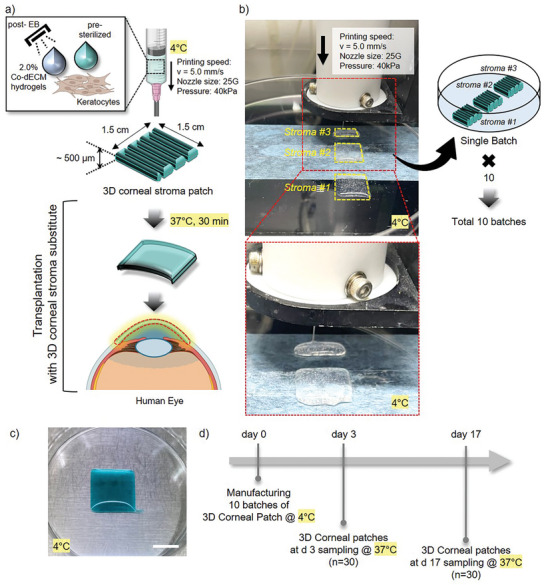
Fabrication process and experimental timeline of 3D corneal stroma patches. a) Schematic of the bioprinting process using pre‐sterilized and EB‐treated Co‐dECM hydrogels (2.0%) with keratocytes, showing the printing parameters (*v* = 5.0 mm s^−1^, 25G nozzle, 40 kPa pressure) to create a 1.5 cm × 1.5 cm stroma patch with ≈500 µm thickness, followed by thermal crosslinking at 37 °C for 30 min and its intended application as a corneal stroma substitute. b) Representative images of the 3D bioprinting process at 4 °C showing the sequential printing of three 3D cornea stroma and final single batch production, with ten batches manufactured in total. c) Photograph of a fabricated 3D corneal stroma patch maintained at 4 °C. Scale bar, 1 cm. (scale bar included). d) Timeline schematic showing the manufacturing of 10 batches at 4 °C on day 0, followed by sampling points on day 3 and day 17 (*n* = 30 patches per timepoint) with patches maintained at 37 °C.

To investigate the robustness of the manufacturing process in maintaining consistent quality, we produced 10 batches of 3D corneal stroma patches. Building on the optimized printing parameters established previously (Figure 3), we successfully manufactured multiple batches of 3D corneal stroma patches (**Figure** **4**). Employing a precisely controlled 3D bioprinting process, each corneal patch was created from bioinks composed of either pre‐sterilized or EB (5 kGy)‐sterilized 2.0% Co‐dECM hydrogels mixed with human keratocytes at a concentration of 5 × 10^6^ cells mL^−1^. These corneal stroma structures were meticulously crafted to precise dimensions of 1.5 cm × 1.5 cm, with a thickness ranging from 500 to 750 µm (Figure 4a,b). In this phase of the study, we manufactured ten batches, each comprising 30 individually printed 3D corneal stroma patches under 4 °C (Figure 4c). This production scale was deliberately chosen to thoroughly assess batch‐to‐batch consistency in terms of both biological viability and structural integrity of the bioprinted 3D corneal stroma patches.

Examining batch‐to‐batch variation through 10 experimental trials is a systematic and rigorous approach in scientific inquiry, particularly when the aim is to ascertain the reproducibility and consistency of a process or product.^[^ ^78^ ^]^ This sample size allowed for robust statistical analysis and provided a comprehensive understanding of the variability inherent in the manufacturing process of 3D corneal stroma patches. Research suggests that increasing the number of experimental replicates enhances the reliability of findings and strengthens the validity of conclusions.^[^ ^79^ ^]^ Furthermore, assessing the batch‐to‐batch consistency across multiple trials enables researchers to identify trends, outliers, and potential sources of variation, thus facilitating further refinement and optimization of manufacturing protocols.^[^ ^80^ ^]^ By conducting trials across 10 batches, this study adhered to the best practices recommended by scientific literature, ensuring robustness in the evaluation of batch‐to‐batch consistency. This approach aligns with the principles of good scientific practice and enhances the credibility of the research findings.^[^ ^81^ ^]^

Moreover, meticulous attention to experimental design and sample size calculation strengthened the scientific rigor of the study, reinforcing confidence in the validity and generalizability of the results.^[^ ^82^ ^]^ Throughout the study, we systematically monitored and analyzed each batch of bioprinted 3D corneal stromal patches under 37 °C to evaluate their viability and maturity at critical time points (days 3 and 17; Figure 4d). This approach not only streamlines the production process but also significantly enhances the reliability and reproducibility of 3D corneal stroma patches. The consistent quality across multiple batches highlights their potential for clinical applications, promising a new frontier in corneal transplantation and regenerative medicine.^[^ ^68^, ^69^ ^]^ Our methodological rigor ensured that each batch adhered to stringent standards, paving the way for future clinical trials and therapeutic implementations.

#### 2.3.3 Impact of the Manufacturing Process on the Variability of Keratocytes in Multiple 3D Corneal Stroma Patches

**Figure 5:**
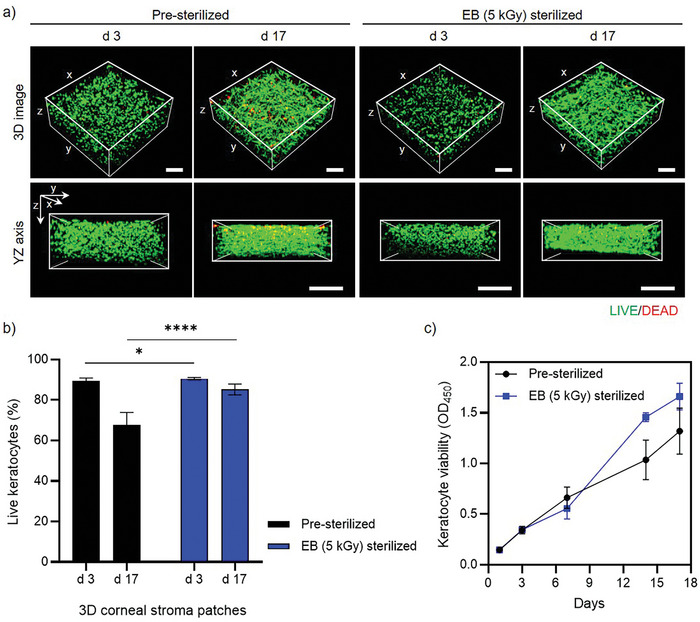
Cell viability analysis of keratocytes in 3D printed corneal stroma patches. a) Representative 3D confocal microscopy images showing LIVE/DEAD staining of keratocytes in Pre‐sterilized and EB (5 kGy) sterilized patches at days 3 and 17. Live cells are shown in green, and dead cells in red, with both top‐view (3D image) and cross‐sectional views (YZ axis) presented. Scale bars, 500 µm. b) Quantitative analysis of live keratocyte percentages in 3D corneal stroma patches at days 3 and 17, with statistical significance indicated by asterisks (*p < 0.05, ****p < *0.0001*). Error bars represent standard deviation. c) Keratocyte viability measurements over 17 days using CCK‐8 assay (OD_450_), comparing pre‐sterilized and EB (5 kGy) sterilized patches. Error bars indicate standard deviation.

To evaluate batch‐to‐batch variation, we rigorously measured the cellular viability and proliferation across different batches of bioprinted 3D corneal stroma patches. Additionally, we assessed the effect of EB (5 kGy) sterilization on bioprinted 3D corneal stroma patches (**Figure** **5**). We analyzed the cell survival capacity within each patch on days 3 and 17 post‐printing across 10 batches (Figure 5a). The results revealed that keratocytes within the 3D corneal stroma patches maintained their viability for 17 days in both the pre‐sterilized and EB (5 kGy)‐sterilized groups (Figure 5b,c). Notably, the keratocyte survival rate was significantly higher in the EB (5 kGy) sterilized group, recording a survival rate of 90.20 ± 3.30%, compared with the control pre‐sterilized group, which had a survival rate of 79.90 ± 14.60% (Figure 5b).

Furthermore, batch‐to‐batch variability was markedly greater in the pre‐sterilized Co‐dECM‐derived printed stroma patch, with a coefficient of variation (CV) of 6.39, in contrast to the lower variability coefficient of 2.92 observed in the EB (5 kGy) sterilized Co‐dECM‐derived printed stroma patch (Figure 5b). The observed CV—6.39 for pre‐sterilized and 2.92 for EB‐sterilized 3D corneal stroma—are crucial for determining their suitability for industrial applications as biopharmaceutical products. According to industry standards, a CV of less than 10% is generally acceptable for most biopharmaceutical processes, indicating good consistency and reliability.^[^ ^83^, ^84^ ^]^ Specifically, the FDA and other regulatory bodies recommend that CV values should ideally be below 10% for intermediate precision and 15% for repeatability in bioanalytical methods.^[^ ^85^ ^]^ A CV of 2.92 for the patches manufactured using EB (5 kGy) sterilized dECM falls well within these acceptable limits, suggesting that the process is suitable for industrial applications. However, a CV of 6.39 for patches manufactured using pre‐sterilized dECM, while higher, may still be acceptable depending on specific regulatory requirements and the criticality of the attribute being measured​.^[^ ^86^ ^]^ The keratocytes in the 3D stroma structures manufactured using EB (5kGy) sterilized dECM demonstrated significantly enhanced survival than those in 3D stroma structures manufactured using pre‐sterilized Co‐dECM (Figure 5b). EB irradiation is more effective at eliminating contaminants because it delivers high‐energy electrons that uniformly penetrate the material, resulting in a more comprehensive reduction of microbial contamination and the breakdown of residual bioinks or crosslinkers.^[^ ^87^ ^]^ These residues, if not properly removed, could be cytotoxic and harm cell viability.^[^ ^88^ ^]^ The precise and deep penetration of EB irradiation makes it especially effective compared to other sterilization methods, which may not achieve the same level of decontamination.^[^ ^89^ ^]^ EB sterilization effectively eradicates potential contaminants without compromising the structural integrity or biological functionality of the bioprinted stroma, thereby providing a more conducive environment for keratocyte proliferation and survival. These detailed analyses also underscore the critical influence of sterilization methods on the quality and consistency of bioprinted 3D corneal stroma patches, highlighting the need for precise control over the manufacturing processes to ensure optimal outcomes in clinical applications.

#### 2.3.4 Impact of EB Sterilization on the Maturity and Reproducibility of Bioprinted 3D Corneal Stroma Patches

**Figure 6:**
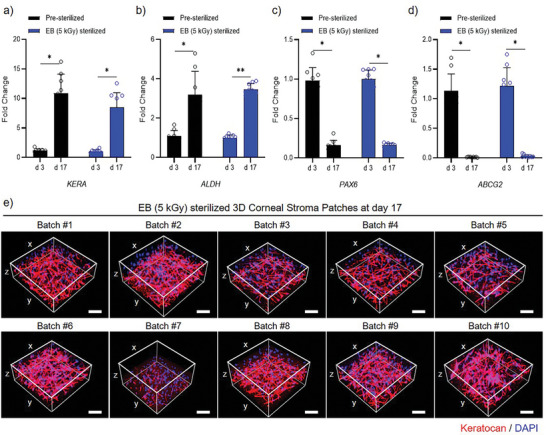
Gene expression analysis and the 3D visualization of corneal stroma patches. a–d) Fold change expression of keratocan (*KERA*), aldehyde dehydrogenase (*ALDH*), paired box 6 (*PAX6*), and ATP‐binding cassette sub‐family G member 2 (*ABCG2*) genes in pre‐sterilized (black bars) and EB (5 kGy) sterilized (blue bars) samples measured at day 3 and day 17. Data points are shown as individual circles with error bars representing standard deviation with statistical significance indicated by asterisks. The error bars represent standard deviation. Asterisks (*p < 0.05, **p < *0.01*) indicate statistical significance. e) 3D confocal microscopy images showing the structure of EB (5 kGy) sterilized corneal stroma patches at day 17 across ten independent batches (#1‐#10). Images display keratocan protein expression (red) and cell nuclei stained with DAPI (blue). The 3D coordinate system (x, y, z) is shown for spatial reference. Scale bars, 500 µm.

The maturity of the 3D corneal stroma patch fabricated through a standardized bioprinting process was systematically evaluated by examining the differentiation of keratocytes over 17 days after printing. Molecular analysis revealed a progressive increase in the mRNA expression of key keratocyte markers (*KERA* for keratocan and *ALDH* for aldehyde dehydrogenase), whereas the expression of embryonic ocular precursor markers (*PAX‐6* and *ABCG2*) was diminished.^[^ ^90^, ^91^, ^92^ ^]^ This pattern suggests normal keratocyte differentiation within the bioprinted 3D constructs (**Figure** **6**). The keratocytes in the 3D corneal stroma patch successfully matured regardless of sterilization, as indicated by the increased expression of *KERA* and *ALDH* and decreased expression of *PAX‐6* and *ABCG2* (Figure 6a,b,c,d). Notably, the absolute gene expression levels of *KERA, ALDH, PAX‐6*, and *ABCG2* were not significantly different (Figure 6a,b,c,d). This consistency underscores the reproducibility and efficacy of the 3D corneal patch production process. Keratocan, a key keratin sulfate proteoglycan, is vital for corneal clarity and structural integrity.^[^ ^93^, ^94^ ^]^ It was successfully expressed in the cytoplasm of keratocytes across various batches of 3D‐printed corneal stroma patches (Figure 6e). These 3D corneal stroma patches, crafted using EB‐sterilized Co‐dECM bioink, showed minimal variation, illustrating that the controlled bioprinting conditions ensured consistent cellular behavior. This consistency is essential for the scalable production of 3D corneal stroma patches, enhancing their safety and availability for transplantation (Figure 6e).

#### 2.3.5 Role of Maturity and Alignment of Keratocytes in Corneal Transparency

**Figure 7:**
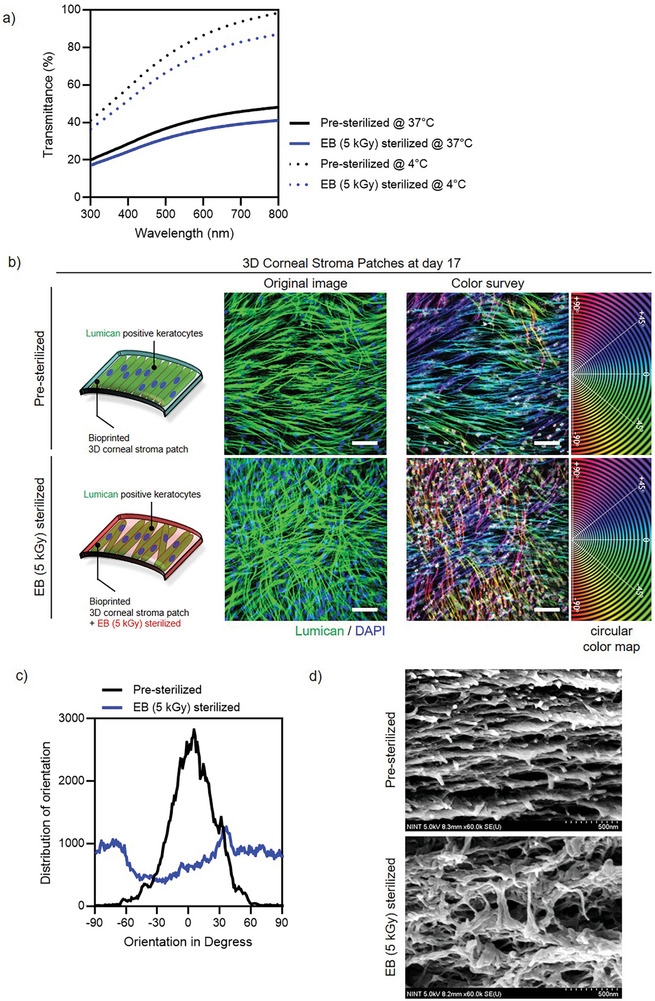
Characterization of 3D‐bioprinted corneal stroma patches before and after electron beam (EB) sterilization. a) Transmittance spectra comparison between pre‐sterilized and EB sterilized samples at different temperatures. The graph shows transmittance (%) as a function of wavelength (nm) from 300–800 nm. Solid lines represent measurements at 37 °C for both pre‐sterilized (black) and EB (5 kGy) sterilized (blue) samples. Dotted lines indicate measurements at 4 °C for pre‐sterilized (black) and EB (5 kGy) sterilized (blue) samples. b) Schematic representation and fluorescence microscopy analysis of 3D corneal stroma patches on day 17. The left panels show illustrations of bioprinted patches with Lumican‐positive keratocytes. Center panels display Lumican (green) and DAPI (blue) staining showing keratocyte distribution and alignment. The right panels present color survey analysis and circular color mapping of the fiber orientation. Scale bars, 100 µm. c) Quantitative analysis of collagen fiber orientation distribution comparing pre‐sterilized (black line) and EB‐sterilized (5 kGy, blue line) patches. d) Scanning electron microscopy (SEM) images revealing the ultrastructural organization of collagen fibers in pre‐sterilized and EB (5 kGy) sterilized patches. Scale bars, 500 nm.

The primary function of the cornea in vision underscores the importance of transparency in corneal substitutes (Bonato^[^ ^95^ ^]^ 2024 Lagali^[^ ^96^ ^]^ 2020). Transparency was assessed by measuring the light transmittance at 550 nm, which revealed a significant reduction in transparency after thermal gelation and sterilization (**Figures** **7**a and S7, Supporting Information). This decrease was attributed to alterations in the hydrogel matrix microstructure induced by EB sterilization (Figure 7a). Moreover, 30 min of gelation at 37 °C further reduced transparency in both pre‐sterilized and EB (5 kGy)‐sterilized corneal stromal patches (Figure 7a).

Lumican plays a pivotal role in maintaining corneal transparency by organizing collagen fibrils and minimizing light scattering.^[^ ^97^, ^98^, ^99^, ^100^, ^101^ ^]^ It is expressed in the ECM of keratocytes, where it regulates collagen alignment to ensure corneal clarity.^[^ ^99^, ^102^ ^]^ In this study, 3D corneal stroma patches prepared using EB (5 kGy)‐sterilized Co‐dECM exhibited disorganized keratocyte alignment affected by disrupted lumican expression patterns (Figure 7b,c,d). Misaligned lumican expression resulted in keratocytes orienting at extreme angles (−90° or +90°) rather than aligning parallelly (0°), which leads to a chaotic collagen arrangement (Figure 7c). This misalignment diminishes the transparency within the visible light range of 300–800 nm (Figure 7a).

Further investigation, supported by SEM imaging, revealed that EB sterilization disrupted the native fibrillar organization of Co‐dECM, particularly lumican‐mediated collagen alignment (Figure 7d). These structural changes are likely caused by the high‐energy electrons of EB sterilization, which modify the ECM chemical bonds, alter its architecture, and affect cellular behavior.^[^ ^103^, ^104^ ^]^ In conclusion, SEM imaging confirmed that EB sterilization disrupted the native fibrillar structure of the Co‐dECM, which includes collagen alignment and lumican distribution, which are critical factors in guiding keratocyte orientation. The high‐energy electrons used in EB sterilization alter ECM protein bonds, which results in changes in collagen fibril organization, lumican distribution, and matrix stiffness. These modifications impair the architectural cues essential for cell alignment and produce the observed isotropic orientation in the EB‐sterilized samples.

Optimizing the sterilization methods that preserve lumican functionality and ECM alignment is crucial for achieving clinical‐grade corneal stromal patches.^[^ ^105^, ^106^ ^]^ Uniform cell and ECM alignment in 3D bioprinted Co‐dECM hydrogels may improve transparency by reducing irregular light scattering. However, excessive alignment can introduce new scattering interfaces, emphasizing the need for the continuous optimization of biofabrication techniques.^[^ ^107^, ^108^, ^109^, ^110^ ^]^ Aligning these innovations with stringent quality controls can help ensure the development of transparent and functional corneal substitutes suitable for transplantation and other clinical applications.^[^ ^36^, ^111^, ^112^ ^]^

#### 2.3.6 Impact of EB Sterilization on Co‐dECM Bioink in Terms of Macrophage Phenotype and Inflammatory Markers

Optimized sterilization methods are crucial for reducing the risk of chronic inflammation and tissue damage in 3D biopharmaceutical products during clinical trials.^[^ ^103^ ^]^ These products must retain their anti‐inflammatory functions to minimize immune rejection after implantation.^[^ ^113^ ^]^

**Figure 8:**
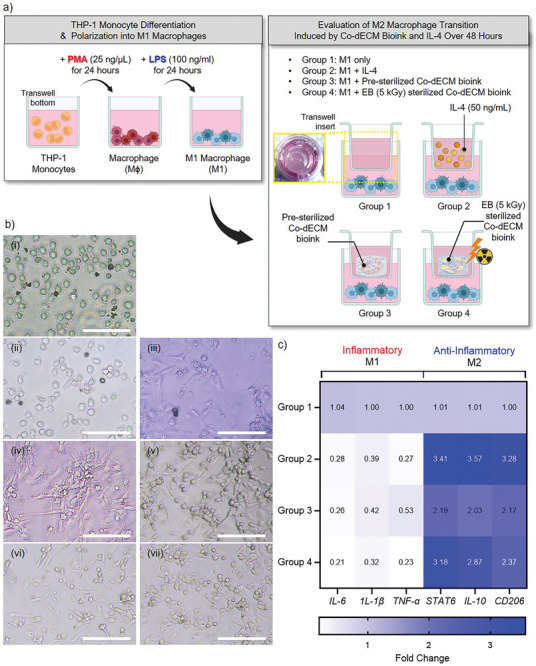
Evaluation of macrophage polarization and inflammatory responses to Co‐dECM bioinks. a) Schematic of the experimental design. Left panel shows THP‐1 monocyte differentiation protocol using PMA (25 ng µL^−1^, 24 h) followed by LPS (100 ng mL^−1^, 24 h) to generate M1 macrophages. Right panel illustrates the four experimental groups for assessing M2 macrophage transition:M1 only (Group 1), M1 + IL‐4 (Group 2), M1 + pre‐sterilized Co‐dECM bioink (Group 3), and M1 + EB‐sterilized Co‐dECM bioink (Group 4), with IL‐4 (50 ng mL^−1^) treatment over 48 h. b) Representative bright‐field microscopy images showing morphological changes in: i) untreated control, ii, iii) differentiation stages, and (iv‐vii) treatment groups after 4 days of culture. Scale bars, 50 µm. c) Heatmap analysis of inflammatory (M1) and anti‐inflammatory (M2) marker expression across experimental groups. The color intensity represents fold change in gene expression for pro‐inflammatory (*IL‐6, IL‐1β, TNF‐α*) and anti‐inflammatory (*STAT6, IL‐10, CD206*) markers, with values normalized to Group 1 (M1 only).

To evaluate the immunomodulatory properties of bioprinted 3D corneal stroma patches, we utilized an in vitro model to investigate macrophage phenotype transitions and inflammatory responses. THP‐1 monocytes were differentiated into macrophages using PMA treatment, followed by Lipopolysaccharide (LPS) stimulation to induce M1 macrophages (**Figure** **8**a).^[^ ^114^ ^]^ To assess M2 polarization, the experimental design included four treatment groups: M1 only (Group 1), M1 with Interleukin‐4 (IL‐4) (Group 2), M1 with pre‐sterilized Co‐dECM bioink (Group 3), and M1 with EB‐sterilized Co‐dECM bioink (Group 4).

Bright‐field microscopy revealed distinct morphological changes during macrophage differentiation and treatment response (Figure 8b). Untreated THP‐1 monocytes exhibited a round morphology (Figure 8b (i)), whereas PMA treatment induced adherence characteristics of macrophages (Figure 8b (ii)). LPS stimulation further promoted spreading, which is indicative of M1 macrophage activation (Figure 8b (iii)). Following exposure to different treatments, the cells displayed varying morphologies, which reflected phenotypic changes in response to bioink treatment.

Gene expression analysis highlighted the differential regulation of inflammatory markers across treatment groups (Figure 8c). Pro‐inflammatory M1 markers, including Interleukin‐6 (IL‐6), *IL‐1β*, and Tumor Necrosis Factor‐Alpha (*TNF‐α*), were significantly reduced in Groups 2–4 compared to the M1‐only control, with the EB‐sterilized Co‐dECM group (Group 4) exhibiting the lowest levels (Figure 8c).^[^ ^114^, ^115^, ^116^, ^117^ ^]^ In parallel, anti‐inflammatory M2 markers, such as signal transducer and activator of transcription 6 (*STAT6*), Interleukin‐10 (*IL‐10*), and *CD206*, were notably upregulated in Groups 2–4, with the IL‐4‐treated group demonstrating the highest expression (Figure 8c).^[^ ^118^ ^]^ Group 4, exposed to EB (5 kGy)‐sterilized Co‐dECM, displayed a balanced inflammatory profile characterized by moderate suppression of M1 markers and a substantial increase in M2 markers, indicating effective immunomodulation.^[^ ^119^, ^120^ ^]^

The results revealed that Co‐dECM possesses inherent immunomodulatory properties (Group 3), as demonstrated by the upregulation of anti‐inflammatory M2 markers (*STAT6, IL‐10, CD206*) and a moderate reduction in pro‐inflammatory M1 markers (*IL‐6, IL‐1β, TNF‐α*) compared to the M1‐only group (Figure 8c). This indicates that Co‐dECM contains bioactive components such as extracellular matrix proteins and glycosaminoglycans, which contribute to its ability to modulate immune responses.^[^ ^121^ ^]^

These immunomodulatory properties are further enhanced by EB sterilization (Group 4), highlighting the dual role of EB sterilization in ensuring sterility and amplifying the functional capabilities of Co‐dECM bioink. The ability of EB (5 kGy)‐sterilized Co‐dECM to promote the transition from pro‐inflammatory M1 macrophages, which are associated with initial inflammation and potential graft rejection, to anti‐inflammatory M2 macrophages, which support tissue repair and regeneration, is particularly significant for tissue engineering applications.^[^ ^11^, ^122^, ^123^ ^]^ By facilitating this M1‐to‐M2 transition, EB (5 kGy)‐sterilized Co‐dECM creates a more favorable immune environment that minimizes inflammation and enhances healing and integration, making it a promising candidate for regenerative medicine and tissue engineering.

These findings have important implications for corneal tissue engineering. Immune rejection is a major challenge in corneal transplantation, and modulating the local immune response can significantly improve outcomes.^[^ ^124^ ^]^ By encouraging a shift toward M2 macrophages, EB‐sterilized Co‐dECM bioinks may reduce the risk of graft rejection, promote faster healing and integration, and support the survival and functionality of transplanted cells. The balanced inflammatory response observed suggests that these bioinks hold great promise for advancing corneal tissue engineering and regenerative medicine.^[^ ^125^, ^126^ ^]^

## 3 Conclusion

This study emphasizes the critical importance of selecting sterilization methods that balance efficacy while preserving the functional qualities of Co‐dECM materials. EB sterilization has emerged as the preferred method, aligning with specific requirements for bioprinting 3D corneal stromal patches by maintaining both the safety and structural integrity of the materials. Unlike traditional methods such as autoclaving, which can significantly impair material functionality, EB sterilization effectively preserves the essential properties of biomaterials. Our findings highlight the complex relationship among sterilization methods, material properties, and corneal transparency. This study revealed that EB sterilization, while effective in maintaining the overall structural integrity, can affect the organization of collagen fibrils and lumican expression patterns. These changes affect keratocyte alignment, and consequently, corneal transparency. The disorganized keratocyte alignment and disrupted lumican expression patterns observed in the EB‐sterilized patches led to a more chaotic collagen arrangement, thereby resulting in reduced transparency within the visible light range. Despite these challenges, EB‐sterilized 3D corneal stromal patches show potential as viable human corneal substitutes. The ability to control and optimize the sterilization process opens new avenues for fine‐tuning the balance between sterility, structural integrity, and optical properties. This advancement can potentially reduce waiting times for patients requiring corneal transplantation while ensuring product safety and efficacy.

This study underscores the necessity for the ongoing refinement of sterilization processes to meet stringent safety and efficacy standards while preserving crucial functional properties such as transparency. Future research should focus on optimizing EB sterilization parameters or exploring complementary techniques to enhance collagen alignment and lumican expression post‐sterilization, which improves the optical properties of bioprinted corneal patches. This comprehensive analysis deepens our understanding of material behavior after sterilization and establishes best practices for preparing biomaterials for medical use. The continuous evolution of 3D bioprinting technology and materials science offers promising avenues for innovation. By leveraging these advancements, there is a prospect for engineering biomaterials that exhibit enhanced durability, functionality, and optical properties, which are capable of withstanding diverse sterilization methodologies without compromising their intrinsic properties.

These advancements have immense potential to revolutionize various medical domains, expand the applicability of advanced 3D bioprinting technologies, and foster transformative breakthroughs in patient care and treatment modalities, particularly in corneal tissue engineering and transplantation.

## 4 Experimental Section

### Preparation of Co‐dECM Materials

The preparation method for Co‐dECM materials was prepared following a detailed protocol.^[^ ^8^ ^]^ Whole corneas were dissected from bovine eyeballs and thoroughly washed with phosphate‐buffered saline (PBS) containing penicillin (100 U mL^−1^) and streptomycin (0.1 mg mL^−1^). The stromal layers were then separated from the corneas and agitated in a solution of 20 mM ammonium hydroxide (NH_4_OH; 4.98 N solution in water, Sigma‐Aldrich, USA) and 0.5% Triton X‐100 (99.9% purity, Bio‐Sesang, Korea) for 4 h. Following this, the tissues were treated in a hypotonic Tris hydrochloride (Tris‐HCl; pH 7.4, Bio‐Sesang, Korea) buffer solution for 24 h and subsequently in a 10 mM Tris‐HCl solution with 1% (v/v) Triton X‐100 at 37 °C for another 24 h, resulting in the formation of Co‐dECM tissues. Tissues were sterilized using a 1% peracetic acid solution (32 wt% in dilute acetic acid, Sigma‐Aldrich, USA) in 50% ethanol for 10 h. After decellularization, Co‐dECM was lyophilized overnight and subsequently ground into a fine powder using liquid nitrogen and a milling machine.

### Immunohistological Analysis of Cryosectioned Corneal Stroma

Glass slides with strong adhesive properties were selected for cryosection preparation. The tissue samples were sectioned at a thickness of 40 µm. For staining, cryosectioned corneal tissues were fixed on slides with 4% paraformaldehyde (PFA) for 10 min at room temperature (RT). The slides were washed three times with 1X PBS‐T (0.05% Tween‐20) for 5 min each at RT and permeabilized with 0.1% Triton‐X 100 in PBS‐T for 10 min at RT. After another three washes with 1X PBS‐T (0.05% Tween‐20) for 5 min each, nonspecific binding was blocked by incubation in 10% bovine serum albumin (BSA) in 1X PBS for 1 h at RT. The samples were incubated overnight (12–16 h) at 4 °C with the primary antibody diluted in 1% BSA in 1X PBS‐T (0.05% Tween‐20). The cells were then washed three more times with 1X PBS‐T (0.05% Tween‐20) for 5 min each at RT and incubated with the secondary antibody diluted in 1% BSA in 1X PBS‐T (0.05% Tween‐20) for 2 h at RT. The procedure was completed with three final washes in 1X PBS‐T (0.05% Tween‐20) for 10 min each at RT, ensuring that the slides remained hydrated and that the samples were handled carefully to avoid damage. Finally, the slides were mounted using ProLong Gold with DAPI (P36931, Invitrogen) for nuclear staining.

### Autoclave Sterilization

Autoclave sterilization was conducted at 121 °C for 15 min, followed by a 60‐min cooldown and drying period. This method uses saturated steam under high pressure to achieve sterilization, thus ensuring the elimination of microbes, including spores.

### EtO Gas Sterilization

EtO gas sterilization involved enclosing each 100 mg sample of Co‐dECM in self‐sealing sterilization pouches. These pouches were placed in a sterilization chamber maintained in an atmosphere of 100% EtO gas at a temperature of 30 °C. The sterilization cycle lasted for 3 h using an HG‐3041E:P system (Ethylene Oxide: Pure) with an EtO concentration of 5 mg mL^−1^. Following the sterilization cycle, the Co‐dECM samples were air‐dried for 12 h to ensure the removal of residual EtO. The EtO gas sterilization was performed at POSCO's Research Institute of Science and Technology (RIST, Pohang City, Republic of Korea).

### EB Sterilization

EB irradiation sterilization involved treating Co‐dECM with a total of 5 kGy electron beam in two stages. An initial dose of 1 kGy was delivered at 15 meters per minute, followed by a second dose of 4 kGy at a slower conveyor speed of 3.75 meters per minute. Irradiation was performed at a current of 1.89 mA (148 mA and 25 Hz). For a total dose of 30 kGy, the conveyor speed was adjusted to 0.5 meters per minute to achieve the required exposure. A recovery process followed this to ensure the integrity and functionality of the biomaterials after sterilization. EB irradiation was conducted at a facility at the Korea Institute of Industrial Technology (KITECH, Yeongcheon‐si, Republic of Korea).

### UV Treatment

The materials were transferred into conical tubes and placed in a laminar flow hood under a UV‐C lamp (254 nm; Portable UV LED lamp, China) to treat the Co‐dECM materials with UV light. The lamp was positioned 10–20 cm above the samples to ensure uniform and consistent exposure. The materials were exposed to UV light for 3 h without disturbing their setup. After treatment, the lamp was turned off, the tubes were removed, and the samples were stored in sterile containers at 4 °C when not used immediately.

### Bioburden Test

The bioburden Test measures the microbial load on pre‐sterilized Co‐dECM.

### Endotoxin Test

Endotoxin levels were evaluated according to the United States Pharmacopoeia 43 – National Formulary 38 (USP 43‐NF38) standards, which include Medical Devices‐Bacterial Endotoxin and Pyrogen Tests, specifically the <85> Bacterial endotoxins test.

### Sterility Test

The sterility of the Co‐dECM after EB sterilization was assessed according to ISO 11737‐2:2019, which specifies microbiological methods for the sterilization of healthcare products.

### dsDNA Digestion

To extract dsDNA from the Co‐dECM, we used the GeneJET Genomic DNA Purification Kit (K0722, Thermo Fisher Scientific, Massachusetts, USA) following the manufacturer's recommended protocol. Each sample, weighing 5 mg, was placed in a 1.5‐ml tube. A digestion solution was prepared by mixing the appropriate volume with Proteinase K, and 200ul of this solution was added to each sample. Subsequently, samples were incubated at 56 °C for ≈2–3 h until fully dissolved. Following the incubation period, 20 µL of RNase A solution was added and incubated for 10 min to prevent RNA contamination. The sample solution was then transferred to a DNA purification column, and a washing buffer was applied to the column. Finally, 200 µL of elution buffer was added to the column matrix and allowed to sit for 2 min to elute the DNA at a concentration of 5 mg/200 µL.

### dsDNA Quantification

For the quantification of dsDNA, we followed the manufacturer's protocol using the Quant‐iT PicoGreen dsDNA assay kits and dsDNA reagents (P7589, Invitrogen). Fluorescence emission intensity was measured at 520 nm using a spectrofluorometer after exciting the DNA samples at 480 nm. This method ensured the sensitive and reliable quantification of the remaining dsDNA content in the samples, thereby enabling an effective evaluation of the extent of decellularization. Residual DNA content was normalized to the dry weight of the tissue. This approach accounts for potential variations in tissue size and composition, ensuring an accurate and standardized assessment of DNA removal across all samples. We aimed to enhance the robustness and reproducibility of our findings by incorporating this normalization step.

### Papain Digestion

To quantify protein and GAG content, we used papain digestion. The papain digestion buffer was prepared by combining 0.1 M sodium phosphate, 5 mM Na_2_‐EDTA, and 5 mM cysteine‐HCl, with the pH adjusted to 6.5 using NaOH. For papain digestion, papain (10 mg mL^−1^) was added at a ratio of 125 µL per 10 mL of total digestion buffer solution and thoroughly mixed. The decellularized extracellular matrix was then subjected to papain digestion using a papain digestion solution (5 mg mL^−1^). This mixture was incubated at 60 °C in an oven for 16 h to ensure complete digestion of the proteins and GAGs present in the matrix.

### GAG Quantification

Sulfated GAGs were detected using the 1,9‐dimethyl methylene blue (DMMB) assay. Initially, a papain‐digested solution containing dECM was prepared and mixed with the DMMB solution. After thorough mixing, the absorbance of the resulting solution was measured at a wavelength of 525 nm using a spectrophotometer.

### Collagen Quantification

Hydroxyproline (HYP) was used as a surrogate marker instead of directly quantifying collagen. HYP, a major constituent of collagen, serves as a convenient proxy for estimating collagen content. A Hydroxyproline Assay kit (Abcam, Catalog No. ab222941, USA) was used to measure collagen following the manufacturer's recommended protocol. A papain‐digested solution containing dECM was transferred into acid‐resistant tubes (Thermo 362800‐0020), with 250‐µL aliquots dispensed. Subsequently, 250 µL of HCl (12N) was added, and the tubes were securely capped using acid‐resistant caps (Thermo 362821‐0111). The digestion process was conducted in a heating block set at 120 °C for complete hydrolysis. Upon completion, the solution was treated with reagent A (Chloramine T + Oxidation Buffer) or B (DMAB + Perchloric acid/isopropanol solution). After incubation for 90 min, the absorbance was measured at 560 nm.

### Preparation of Co‐dECM Hydrogel

To prepare the Co‐dECM hydrogel, 0.2 g of powdered Co‐dECM was dissolved in 10 mL of a 0.5 M acetic acid solution (Merck, USA). This solution was further supplemented with 0.02 g of pepsin (Sigma‐Aldrich, USA) to facilitate the removal of telopeptides from the collagen molecules. The digestion process was carried out over 4 days to ensure the complete breakdown of the materials. Following the digestion, the resultant 2.0% Co‐dECM was then filtered through a 100‐µm mesh to remove any undigested particles. The pH of the dECM solution was adjusted to a neutral range of 7.0–7.4 using a 10 M sodium hydroxide (NaOH) solution (Sigma‐Aldrich, USA) to obtain a hydrogel. All steps in the preparation process were conducted at a controlled temperature of 4 °C to preserve the integrity of the hydrogel components.

### Rheological and Mechanical Characterization

The rheological and mechanical properties of Co‐dECM‐based hydrogels, including sterilized variants, were assessed using an advanced rheometric expansion system (TA Instruments, USA) with a 250 µm gap set between a 20‐mm‐diameter geometry and 250 µL of the material. Viscosity was measured by varying the shear rate from 0.1 to 1000 s^−^¹ at 15 °C (*n* = 3), while yield and flow points were determined by analyzing the complex modulus under shear strain from 0.1%–1000% at the same temperature. Gelation kinetics were evaluated using a temperature sweep oscillatory test, with the temperature being increased from 4 to 37 °C, and compressive modulus was measured under shear strain ranging from 0.1% to 1000% at 15 °C (*n* = 3). Finally, shear recovery properties were tested by cyclically altering the shear strain between 10% and 1000% at 4 °C.

### Rheological and mechanical characterization

The rheological and mechanical properties of Co‐dECM‐based hydrogels, including sterilized variants, were assessed using an Advanced Rheometric Expansion System (TA Instruments, USA) with a 250 µm gap set between a 20‐mm‐diameter geometry and 250 µL of the material. Viscosity was measured by varying the shear rate from 0.1 to 1000 s⁻¹ at 15 °C (*n* = 3), while yield and flow points were determined by analyzing the complex modulus under shear strain from 0.1 to 1000% at the same temperature. Gelation kinetics were evaluated using a temperature sweep oscillatory test, with temperatures increased from 4 to 37 °C, and compressive modulus was measured under shear strain ranging from 0.1 to 1000% at 15 °C (*n* = 3). Last, shear recovery properties were tested by cyclically altering shear strain between 10 and 1000% at 4 °C.

### Compressive Test

Compressive testing was performed using a MicroTester G2 (CellScale, Canada). Cylindrical thermally crosslinked Co‐dECM samples, each with a diameter and height of 5 mm, were placed between the testing anvils. Compression was applied using a microbeam (Diameter: 0.4500 mm, resolution: 34.52 µN) with a 6 × 6 mm stainless steel plate affixed to its end. The test utilized a displacement‐controlled mode with the compression rate set at 4% strain per minute.

### Circular Dichroism Spectrometry

For circular dichroism (CD) spectroscopy, 1 mg of digested dECM was dissolved in 1 mL of 7 mM phosphate buffer (pH 7.4) and stirred overnight at 4 °C to ensure complete solubilization. Insoluble particles were removed by gentle centrifugation, and the resulting supernatant was used for CD measurements. The protein concentration was adjusted to 0.1 mg mL^−1^. The prepared solution was transferred into a quartz cuvette with a 0.1 cm path length (Jasco SPA‐1) for the analysis. The CD spectra were recorded using a Jasco J‐815 spectropolarimeter (Jasco GmbH) equipped with a recirculating and a Jasco PTC‐423S Peltier temperature controller (Jasco GmbH) under a nitrogen atmosphere at 25 °C. The measurements were conducted in the far‐UV range, scanning wavelengths from 240–190 nm at 0.2 nm intervals, with a scanning speed of 5 nm min^−1^ per sample. Phosphate buffer (7 mM) was used as a blank and its signal was subtracted from the spectra. The mean residue ellipticity (MRE; deg·cm^2^ dmol^−1^) was calculated from the raw CD data (millidegrees, m°) using

(1) $$\mathrm{MRE}=\frac{m^{\circ }}{M\cdot \left(10\cdot L\cdot C\right)}$$

where *M* represents the average molecular weight of the amino acid (120 g mol^−1^), *L* represents the path length of the cuvette (0.1 cm), and *C* represents the protein concentration (g L^−1^).

### Fabrication of 3D Corneal Stroma Patch

The 3D corneal stroma patch was fabricated by encapsulating commercially available human keratocytes (ScienCell, Catalog No. 6520) at passage two or three, at a concentration of 5 × 10⁶ cells mL^−1^. The human‐derived cells used in this study were purchased from ScienCell, a certified commercial provider. According to the documentation of supplier, the cells were obtained in compliance with ethical guidelines and regulatory approvals, ensuring proper donor consent and adherence to ethical standards. As the cells were commercially sourced, no direct human subject involvement was required for this study. Furthermore, the human keratocytes were mixed with hydrogels prepared using either EB‐sterilized or pre‐sterilized Co‐dECM to create a bioink. The cell encapsulation process was performed in an ice bath to maintain the optimal conditions for cell viability and hydrogel stability. A microextrusion‐based 3D bioprinting system (3DXPrinter, T&R Biofab, Republic of Korea) was employed for 3D bioprinting. The printing parameters were set as follows: printing speed of 5.0 mm ^−1^s, using a 25G cylindrical printing nozzle, and pressure of 40 kPa. The printed 3D corneal stromal patches were incubated at 37 °C in a humidified atmosphere containing 5% CO_2_. The 3D stromal structures were cultured in a fibroblast medium (FM, ScienCell, Catalog No. 2301, USA) prior to sampling. This process ensured that keratocytes were well‐incubated for optimal growth and integration within the fabricated 3D corneal stromal patch.

### Cell Viability Analyses

A live/dead assay was used to assess the viability of keratocytes in a 3D corneal stromal patch. This assay utilized a live/dead kit (Thermo Fisher Scientific) containing calcein‐AM (green, live) and ethidium homodimer (red, dead) in accordance with the manufacturer's instructions. For each group, three random field‐of‐view images were captured, and the proportion of dead cells was quantified and expressed as a percentage of the total cells. Cell metabolic activity was measured using the Cell Counting Kit‐8 (CCK‐8; Chromo‐CKTM, Chromogen, Republic of Korea). After culturing the 3D corneal stroma patch for 3 and 7 days, the CCK‐8 solution was added and incubated for 4 h. The absorbance of the resulting supernatant was measured at 450 nm using a Multiskan SkyHigh Microplate Spectrophotometer (Thermo Fisher Scientific, USA). Measurements were repeated and aggregated from multiple independent experiments.

### RNA Extraction, Reverse Transcription, and qPCR Analyses

Total RNA was extracted using a GeneJET RNA Purification Kit (Thermo Fisher Scientific) according to the manufacturer's instructions. Complementary DNA was synthesized using a cDNA synthesis kit from the same manufacturer. The relative gene expression levels were measured using SYBR Green on a StepOnePlus Real‐Time PCR System (Applied Biosystems). The results were quantified using the 2^−ΔΔCt^ method, with normalization against *GAPDH* as a housekeeping gene. Independent experiments were conducted as specified in each figure legend to analyze the quantitative polymerase chain reaction (qPCR) graphs. Primers were designed based on sequences from the National Center for Biotechnology Information (NCBI) reference sequence database (**Table** **2**).

**Table 2:** List of primer sequences.

Gene	Sequence (5′→3′)	
***KERA***	Forward	GCCTCCAAGATTACCAGCCAA
	Reverse	ACGGAGGTAGCGAAGATGAGGT
***ALDH***	Forward	CGCTCCTGATGCAAGCATGGAAGC
	Reverse	CTCCCAACAACCTCCTCTATGGCT
***PAX6***	Forward	AGATGAGGCTCAAATGCGAC
	Reverse	GTTGGTAGACACTGGTGCTG
***ABCG2***	Forward	TGCAACATGTACTGGCGAAGA
	Reverse	TCTTCCACAAGCCCCAGG
***IL‐6***	Forward	GCCACTCACCTCTTCAGAACG
	Reverse	TTTCACCAGGCAAGTCTCCTC
***IL‐1β***	Forward	AGCCATGGCAGAAGTACCTG
	Reverse	CCTGGAAGGAGCACTTCATCT
***TNF‐α***	Forward	TCTGGGCAGGTCTACTTTGGG
	Reverse	GAGGTTGAGGGTGTCTGAAGG
***STAT6***	Forward	ATGGGGCAACAGAAAAGATG
	Reverse	GCACAGAAGACAGCAGCAAG
***IL‐10***	Forward	TCAAGGCGCATGTGAACTCC
	Reverse	GATGTCAAACTCACTCATGGCT
***CD206***	Forward	AAGGCGGTGACCTCACAAG
	Reverse	AAAGTCCAATTCCTCGATGGTG
***GAPDH***	Forward	GGGTTTCCCGTTGATGACC
	Reverse	GTTACCAGGGCTGCCTTCTC

### Immunofluorescence Staining

The bioprinted 3D corneal stromal structures were subjected to immunofluorescence staining. Fixation was achieved by overnight immersion in ice‐cold methanol at 4 °C. Following fixation, the 3D corneal stromal patches were washed thrice with cold PBS and permeabilized with 0.1% (v/v) Triton X‐100 (Sigma‐Aldrich, USA) in PBS for 10 min. Subsequently, a blocking solution consisting of 1% (w/v) bovine serum albumin in PBS was applied to the 3D stromal structure. After blocking, the samples were washed with 0.1% PBST and incubated with primary antibodies against lumican (1:100; Abcam, Catalog No. ab168348, USA) and keratocan (1:10; Abcam, Catalog No. ab230175, USA) for 1 h at room temperature (25 °C). Excess primary antibodies were removed by washing the samples thrice with 1% PBST, followed by incubation with Alexa Fluor secondary antibodies (Invitrogen, Germany) for 1 h. After incubation, the samples were washed with PBS, and the 3D corneal stroma patches were mounted using VECTASHIELD Antifade Mounting Medium with DAPI (H‐1200‐10). The samples were observed under a confocal microscope (Nikon Eclipse Ti2 confocal microscope, Nikon, Japan, and FluoView FV1000, Olympus, Japan). The captured images were processed and analyzed using Nikon NIS‐Elements software and Olympus FluoView31S‐SW software.

### Image Analysis using OrientationJ

ImageJ was used with the OrientationJ plugin to analyze confocal microscopy images. The images were converted to 8‐bit grayscale, and contrast was adjusted as needed. The OrientationJ plugin was then used to select the region of interest (ROI) and configure parameters for scale and window size. After running the analysis, the plugin provided orientation data as a histogram or color‐coded map, which was saved for further analysis.

### Transmittance Examinations

The transmittance spectra of the 3D corneal patches on day 17 were measured using a multimode microplate reader (Varioskan LUX; Thermo Fisher Scientific, USA). Portions of the 3D corneal samples were placed in a 96‐well plate, and the absorbance spectra were recorded across a wavelength range of 300–800 nm at a resolution of 1 nm.^[^ ^127^ ^]^ Transmittance (%) was calculated using

(2) $$\mathrm{Transmittance}\cdot \left(\%\right)=A=\log_{10}\left(1/T\right)=\log_{10}\left(I_{0}/I\right)$$

where *I_0_* and *I* represent the incident and transmitted light intensities, respectively.

### Scanning Electron Microscopy

A step‐by‐step protocol was followed to prepare Co‐dECM and EB‐sterilized Co‐dECM samples for SEM analysis and to ensure the preservation of internal structures and enhance imaging quality. First, 2.0% (w/v) Co‐dECM was prepared and crosslinked. For fixation, the specimens were immersed in 2.5% glutaraldehyde solution and incubated at room temperature for 1 h. Following fixation, the samples underwent lyophilization, which is a rapid freeze–drying process, for 3 days to preserve their morphology. Then, the lyophilized samples were sectioned into ≈2 mm pieces to facilitate imaging. The specimens were sputter‐coated with gold using an Eiko IB sputter coater (Kyoto, Japan) to improve the conductivity and reduce charging artifacts. Finally, the prepared samples were examined using a scanning electron microscope at an acceleration voltage of 10 kV. This optimized protocol enabled the detailed observation of the internal structures of the Co‐dECM specimens while maintaining their morphological integrity through chemical fixation and lyophilization. The gold coating further enhanced conductivity, ensuring high‐quality SEM imaging.

### Evaluation of Immunogenicity in Pre‐Sterilized and EB‐Sterilized Co‐dECM Bioinks

The evaluation process spanned 17 days, during which human keratocytes matured in both the EB‐sterilized and pre‐sterilized Co‐dECM bioinks. Simultaneously, THP‐1 human monocytes (TIB‐202) were cultured in RPMI medium (Gibco, Grand Island, NY) supplemented with 10% fetal bovine serum (FBS) and 1% penicillin/streptomycin at 37 °C with 5% CO_2_. The differentiation of THP‐1 monocytes (1 × 10⁶ cells mL^−1^) into macrophages was induced by treating cells with 25 ng µL^−1^ phorbol myristate acetate (PMA, Sigma‐Aldrich, USA) on the bottom of Transwell inserts (Corning, USA) for 24 h. Subsequently, macrophages were stimulated with 100 ng mL^−1^ lipopolysaccharide (LPS; Sigma‐Aldrich, USA) for 24 h to induce M1 polarization. Subsequently, the M1 macrophages were introduced into a system containing Co‐dECM bioinks (pre‐sterilized or EB‐sterilized). Co‐culturing was performed by combining macrophage‐containing bottom wells with corneal patch–containing inserts in a 1:1 mixture of RPMI and FM. This system was maintained for 48 h to assess macrophage polarization. As a positive control, IL‐4 (Sino Biological, China) treatment was applied for 48 h to induce M2 macrophage transition, which serves as a benchmark for evaluating the immunomodulatory effects of the Co‐dECM bioinks.

### Statistical Analyses

The experimental results were rigorously analyzed across multiple trials, with specific trial numbers provided in the figure legends. Quantitative data are expressed as mean ± standard deviation (S.D.). Statistical comparisons were performed using *t*‐tests, as appropriate. GraphPad Prism 10 (Systat Software USA) was used for statistical analysis.

## Conflict of Interest

The authors declare no conflict of interest.

## Author Contributions

Jungbin Yoon conceptualized the technique, conducted all experiments, thoroughly analyzed the data, drafted the manuscript, and performed revisions. Yeon‐ju Lee and Ju Young Park provided essential raw materials (e.g., porcine cornea). Minji Kim contributed to discussions on biochemical and mechanical studies during major revisions. Jinah Jang, as the supervisor, oversaw the entire study, including data analysis and manuscript writing, and secured funding.

## Supporting information



Supporting Information

## Data Availability

The data that support the findings of this study are available in the supplementary material of this article.
