# Supplementary material for: Enhanced Bioprinting of 3D Corneal Stroma Patches with Reliability, Assessing Product Consistency and Quality through Optimized Electron Beam Sterilization
Source: Adv Healthc Mater. 2025 Feb 10;14(9):2403118. doi: 10.1002/adhm.202403118 (PMC11973947; doi:10.1002/adhm.202403118)
Supplement: Supplementary file 1 — Supporting Information [file ADHM-14-0-s001.docx]

Supporting Information

Enhanced Bioprinting of 3D Corneal Stroma Patches with Reliability, Assessing Product Consistency and Quality through Optimized Electron Beam Sterilization

Jungbin Yoon, Yeon-ju Lee, Minji Kim, Ju Young Park, Jinah Jang*


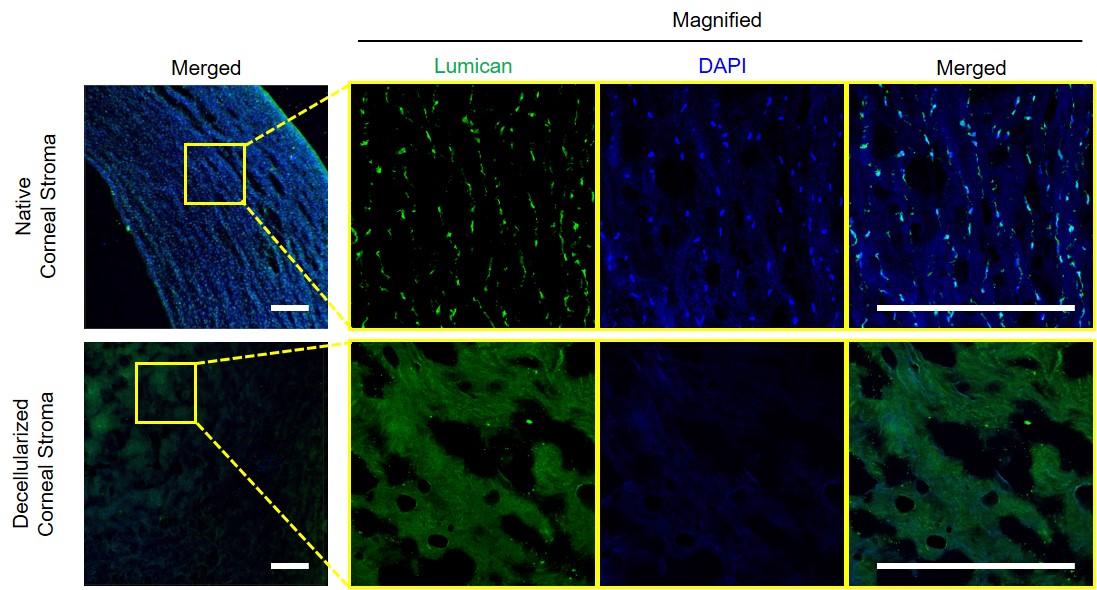


**Figure S1.** Comparison of native and decellularized corneal stroma architecture. Representative immunofluorescence images showing the structural organization of native and decellularized corneal stroma. Left panels display merged images with yellow boxes indicating magnified regions. Right panels show individual and merged channels of the magnified areas with Lumican (green) and DAPI (blue) staining. Native corneal stroma exhibits aligned keratocytes and organized collagen structure, while decellularized stroma maintains the overall architecture with visible ECM components; however, it lacks cellular content. Scale bars, 500 μm.


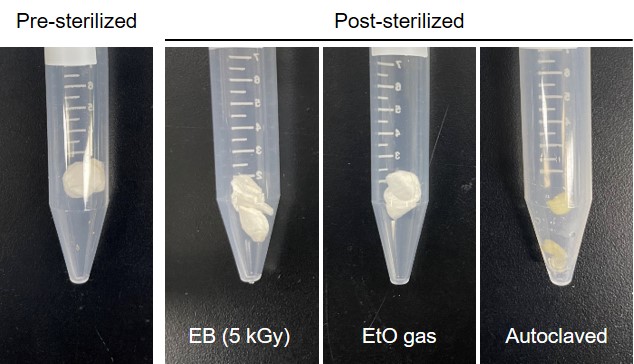


**Figure S2.** Comparison of different sterilization methods on sample materials. The image displays four test tubes demonstrating the effects of various sterilization techniques: pre-sterilized control (leftmost), electron beam radiation at 5 kGy (EB), ethylene oxide gas treatment (EtO gas), and autoclave sterilization (rightmost). Visual changes in the sample material can be observed across different sterilization methods, with each technique showing distinct effects on the physical appearance of the contents. The pre-sterilized sample serves as a baseline for comparing the structural modifications induced by each sterilization process.


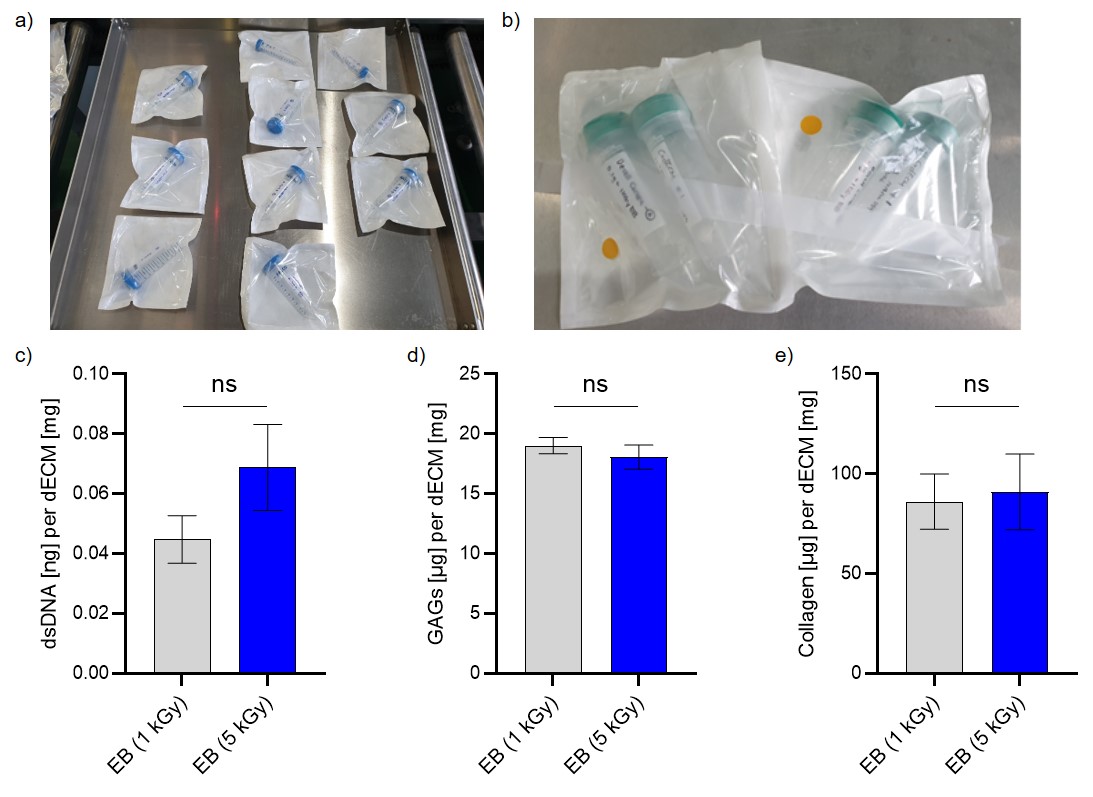


**Figure S3.** Analysis of sterilization methods and their effects on decellularized extracellular matrix (dECM) components. a) Individually packaged syringes containing dECM samples were prepared for sterilization processing. b) Vacuum-sealed packages of dECM samples with indicator dots for sterilization verification. c-e) Quantitative comparison of key ECM components between samples treated with electron beam (EB) radiation at 1 kGy versus 5 kGy: c) dsDNA content showing no significant difference (ns) between radiation doses, d) glycosaminoglycans (GAGs) content remaining stable across both conditions and e) collagen content demonstrating consistent preservation regardless of radiation intensity. Error bars represent standard deviation, and "ns" indicates no statistically significant difference between groups.


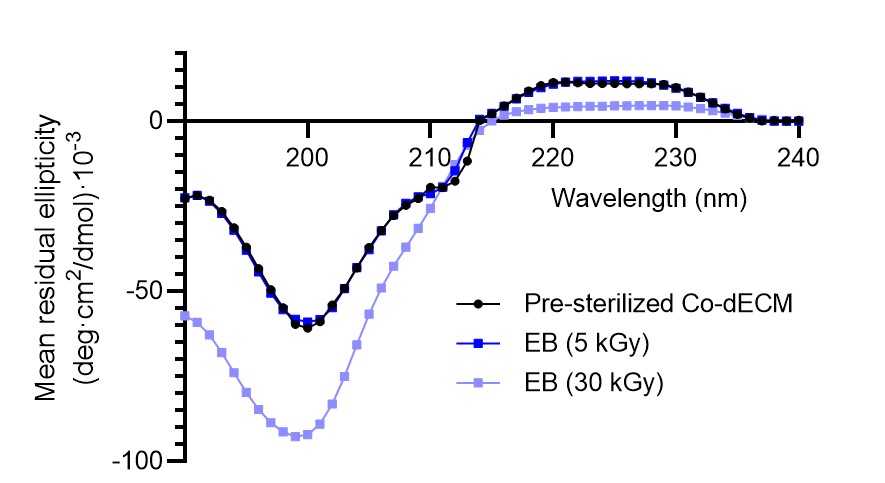


**Figure S4.** Protein structure analysis and sterilization effects on Co-dECM. The graph shows circular dichroism (CD) spectra of Co-dECM samples under different treatments, as measured in the 200–240 nm range. Conditions include pre-sterilized Co-dECM (black circles), electron beam (EB) at 5 kGy (dark blue squares), and 30 kGy (light blue squares). The 190–240 nm range analyzes a protein secondary structure, with a negative peak at 208–210 nm and a positive shoulder at 222 nm, indicating α-helical content. The positive signal near 222 nm reflects triple-helical structures, whereas a deeper negative peak near 200 nm indicates increased random coil formation, particularly at 30 kGy.


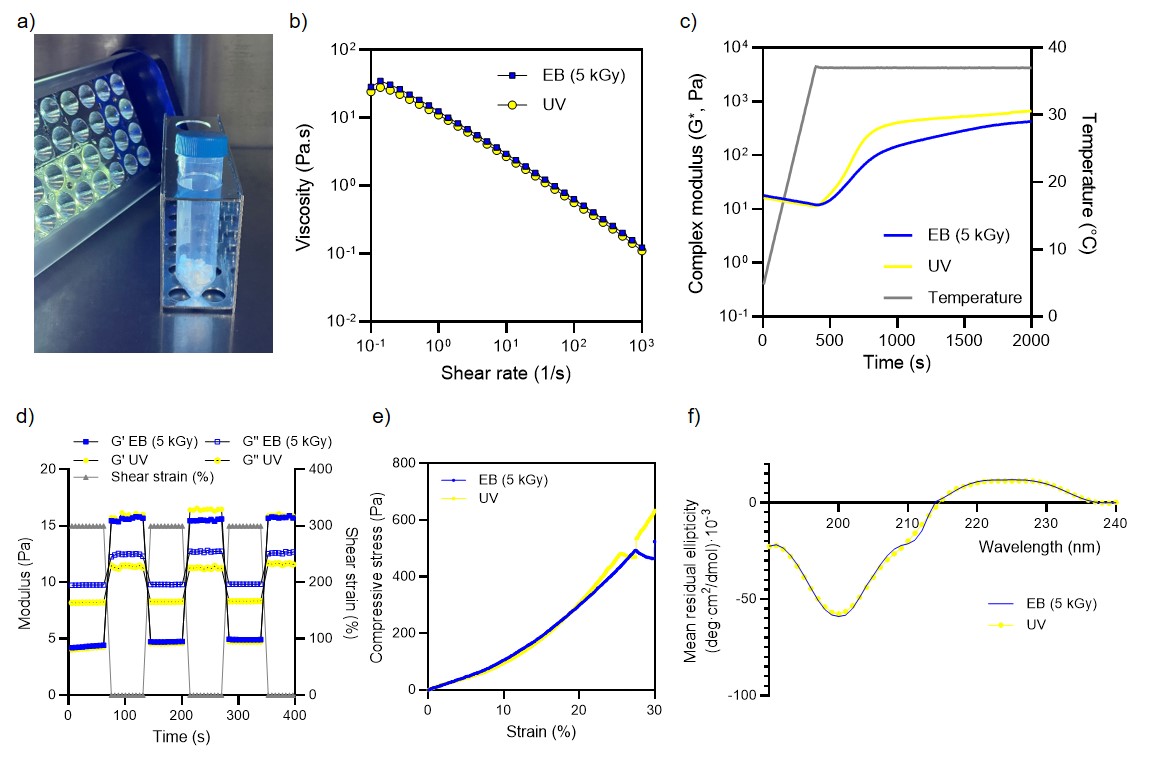


**Figure S5.** Rheological and structural characterization of sterilized Co-dECM samples. a) Sample preparation setup showing UV treatment for Co-dECM samples. b) Viscosity measurements comparing electron beam (EB, 5 kGy) and UV sterilized samples across different shear rates. c) Temperature-dependent complex modulus (G*) analysis showing the gelation kinetics of EB and UV-treated samples over time. d) Oscillatory rheology measurements displaying storage modulus (G'), loss modulus (G"), and shear strain for both sterilization methods. e) Compressive stress–strain curves demonstrating mechanical properties up to 30% strain. f) Circular dichroism spectra comparing secondary structure preservation between EB and UV sterilized samples across 200–240 nm wavelength range. The data collectively demonstrates comparable rheological and structural properties between EB and UV sterilization methods, with both maintaining essential biomaterial characteristics.


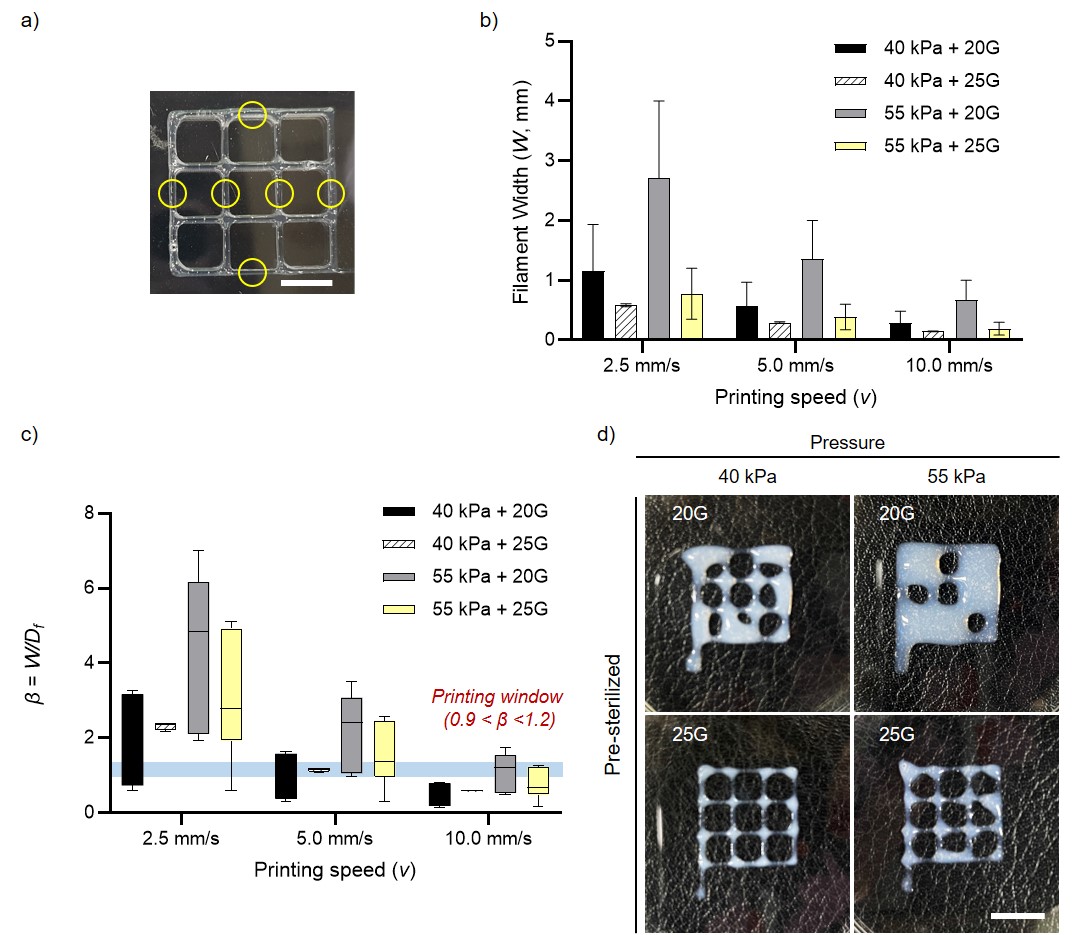


**Figure S6.** Optimization of bioprinting parameters and structural characterization. a) Representative image of printed structure with yellow circles indicating the six measurement points for filament width analysis. Scale bar, 5 mm. b) Quantitative analysis of filament width across different printing speeds (2.5, 5.0, and 10.0 mm/s) under varying pressure conditions (40 and 55 kPa) and needle gauges (20G and 25G). c) Beta (*β*) value analysis shows the relationship between filament width and theoretical diameter (*W/D_f_*) across different printing speeds, with the optimal printing window highlighted in blue (*0.9 < β < 1.2*). d) Photographic comparison of pre-sterilized printed structures after thermal crosslinking, which demonstrates the effects of different pressure conditions (40 and 55 kPa) and needle gauges (20G and 25G) at a 5 mm/s printing speed. Scale bar, 1 cm.


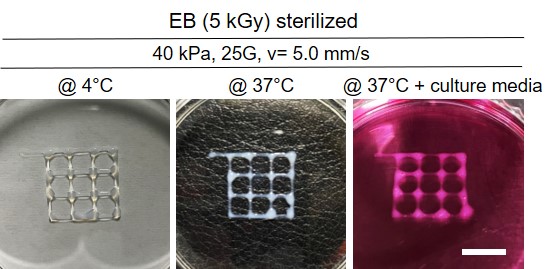


**Figure S7.** Characterization of electron beam (EB) sterilized bioprinted structures. Representative optical images of bioprinted grid structures (40 kPa, 25G needle and printing speed 5.0 mm/s) after EB sterilization (5 kGy) under different conditions: at 4 °C (left), at 37 °C (middle), and 37 °C with culture media (right). Scale bar, 1 cm.
